# Genome-Wide DNA Methylation Scan in Major Depressive Disorder

**DOI:** 10.1371/journal.pone.0034451

**Published:** 2012-04-12

**Authors:** Sarven Sabunciyan, Martin J. Aryee, Rafael A. Irizarry, Michael Rongione, Maree J. Webster, Walter E. Kaufman, Peter Murakami, Andree Lessard, Robert H. Yolken, Andrew P. Feinberg, James B. Potash, GenRED Consortium

**Affiliations:** 1 Department of Pediatrics, Stanley Division of Developmental Neurovirology, Bloomberg School of Public Health, Johns Hopkins University, Baltimore, Maryland, United States of America; 2 Department of Oncology, Bloomberg School of Public Health, Johns Hopkins University, Baltimore, Maryland, United States of America; 3 Epigenetics Center, Bloomberg School of Public Health, Johns Hopkins University, Baltimore, Maryland, United States of America; 4 Division of Molecular Medicine, Department of Medicine, Bloomberg School of Public Health, Johns Hopkins University, Baltimore, Maryland, United States of America; 5 Department of Biostatistics, Bloomberg School of Public Health, Johns Hopkins University, Baltimore, Maryland, United States of America; 6 Center for Genetic Disorders of Cognition and Behavior, Kennedy Krieger Institute, Baltimore, Maryland, United States of America; 7 Uniformed Services University of the Health Sciences, Bethesda, Maryland, United States of America; 8 Maryland Psychiatric Research Center, University of Maryland School of Medicine, Baltimore, Maryland, United States of America; 9 Department of Psychiatry, University of Iowa, Iowa City, Iowa, United States of America; RIKEN Brain Science Institution, Japan

## Abstract

While genome-wide association studies are ongoing to identify sequence variation influencing susceptibility to major depressive disorder (MDD), epigenetic marks, such as DNA methylation, which can be influenced by environment, might also play a role. Here we present the first genome-wide DNA methylation (DNAm) scan in MDD. We compared 39 postmortem frontal cortex MDD samples to 26 controls. DNA was hybridized to our **C**omprehensive **H**igh-throughput **A**rrays for **R**elative **M**ethylation (CHARM) platform, covering 3.5 million CpGs. CHARM identified 224 candidate regions with DNAm differences >10%. These regions are highly enriched for neuronal growth and development genes. Ten of 17 regions for which validation was attempted showed true DNAm differences; the greatest were in *PRIMA1*, with 12–15% increased DNAm in MDD (p = 0.0002–0.0003), and a concomitant decrease in gene expression. These results must be considered pilot data, however, as we could only test replication in a small number of additional brain samples (n = 16), which showed no significant difference in *PRIMA1*. Because PRIMA1 anchors acetylcholinesterase in neuronal membranes, decreased expression could result in decreased enzyme function and increased cholinergic transmission, consistent with a role in MDD. We observed decreased immunoreactivity for acetylcholinesterase in MDD brain with increased *PRIMA1* DNAm, non-significant at p = 0.08.

While we cannot draw firm conclusions about *PRIMA1* DNAm in MDD, the involvement of neuronal development genes across the set showing differential methylation suggests a role for epigenetics in the illness. Further studies using limbic system brain regions might shed additional light on this role.

## Introduction

Family studies show that siblings of probands with major depressive disorder (MDD) have about a three-fold elevated risk of illness, while the estimated heritability of MDD from twin studies is about 37% [1]. The modest level of heritability suggests that the DNA sequence does not fully explain the variability in susceptibility to this illness. Indeed, genome-wide association studies have not yet definitively identified variants implicated in MDD, though some intriguing results have been reported [2].

There are at least two other major kinds of explanations for this variation in susceptibility. One is that environmental factors such as stressful life events play a significant role in triggering MDD [3], and another is that epigenetic factors are involved. These may be interdependent as the environment may cause epigenetic changes. In an animal model of early-life stress characterized by reduced maternal care, epigenetic changes, including increased DNA methylation (DNAm), were seen in the promoter region of the glucocorticoid-receptor gene, and these persisted into adulthood, where they correlated with disruption of the hypothalamic-pituitary-adrenal axis [4]. Analogously, DNA from postmortem hippocampus obtained from suicide victims with a history of childhood abuse, also showed increased DNAm in the human version of the same gene [5].

Epigenetics, which has been frequently implicated in cancers [6], has also been implicated in brain diseases, such as Rett syndrome [7] and fragile X syndrome [8]. There is now ample evidence that DNAm plays a critical role in brain development and function. One study found that abnormally hypomethylated CNS neurons were impaired functionally and were selected against in postnatal development [9]. We have shown that DNAm signatures distinguished three brain regions—cortex, cerebellum, and pons [10]. A role for epigenetics in MDD and other psychiatric disorders has been suggested based on factors such as the lack of complete concordance in monozygotic twins, the onset of illness in adolescence or adulthood rather than childhood, the often episodic nature of the illnesses, and the apparent relationship to environmental factors, including stress [11].

**Figure 1:**
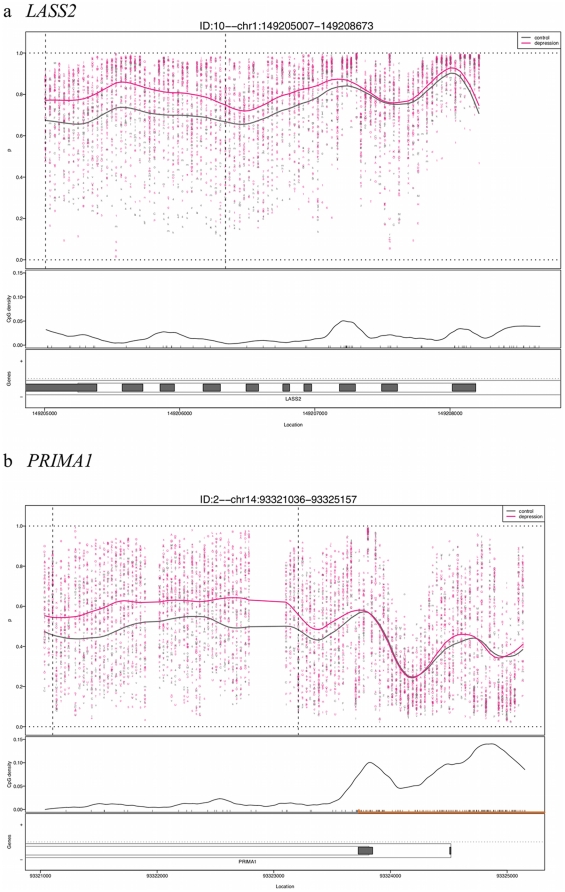
Examples of CHARM results for two of the regions showing greatest DNAm differences between MDD cases and controls. The plots show percent methylation versus genomic location with each point representing the methylation level of an individual sample for a given probe. The curve represents averaged smoothed percent methylation values. The locations of CpG dinucleotides are indicated with black tickmarks on the X-axis. CpG density was calculated across the region using a standard density estimator and is represented by the smoothed black line. The location of the CpG island is denoted on the X-axis as an orange line. Gene annotation is indicated, showing *LASS2* in (a) and *PRIMA1* in (b). The thin outer grey line represents the transcript, while the thin inner lines represent a coding region. Filled in grey boxes represent exons.

**Table 1:** Stanley Medical Research Institute MDD and control brain samples.

	N	Age	M	F	PMI (hr)	pH	% suicide	L	R
Control	27	48.2±10.5	23	4	26.5±15.5	6.6±0.5	0	12	15
MDD	39	44.6±10.6	28	11	44.5±32.8	6.6±0.5	53.8	21	18

There are several examples of epigenetic variation in candidate MDD genes and in DNA treated with medications used for MDD. For example, early life adversity increased DNAm in *Bdnf* in rats [12]. Valproate [13], used to treat bipolar depression, and haloperidol [14], used for psychotic depression, as well as the antidepressants imipramine [15], tranylcypromine [16], and fluoxetine [17] have been shown to induce epigenetic changes in rodent brain. Further, administration of a histone deacetylase inhibitor, sodium butyrate, produces an antidepressant effect in an animal model [18].

Despite the availability of an essentially complete genome sequence for several years, understanding of the methylome has progressed more slowly, largely due to limitations in technology affecting sensitivity, specificity, throughput, quantitation, and cost among the previously used detection methods. Microarray-based methods can interrogate much larger numbers of CpGs than other approaches. One study to date has reported on a genome-wide DNAm study in psychiatric disorders, demonstrating differences in the 4–9% range between DNA from bipolar disorder or schizophrenia brain samples vs. controls [19]. This study used the methylation-sensitive restriction enzymes *Hpa*II and *Mcr*BC to prepare DNA, which they hybridized to a 12,192 CpG-island microarray.

We have similarly used a methylation-sensitive restriction enzyme-based method focused on *Mcr*BC, though we have implemented it on a microarray platform (CHARM), which is not biased towards CpG islands, but rather has features chosen agnostically based on high CpG density. We have shown that CHARM robustly distinguishes tissue types based on differential DNAm profiles, and can also discriminate between colon cancer and normal colon tissues [20].

We have now used CHARM analysis to study genome-wide DNAm variation in 39 MDD and 26 control brains. Here we report results of this experiment and of follow-up pyrosequencing experiments to attempt to validate the initial findings and to correlate DNAm differences with gene expression. While these data should be followed up on a much larger replication set, the absence of large DNAm differences in the brains of MDD patients is itself important in considering the epigenetic hypothesis. These results suggest that if DNAm plays a role in MDD, the most critical target may not be the frontal cortex, but other regions, such as hippocampus and amygdala, key components of the limbic system, in which epigenetic changes have been shown to influence cognitive and behavioral phenotypes [21].

## Materials and Methods

### Ethics Statement

The Johns Hopkins University IRB approved all research involving human participants. Subjects gave written informed consent under the IRB-approved protocol.

**Figure 2:**
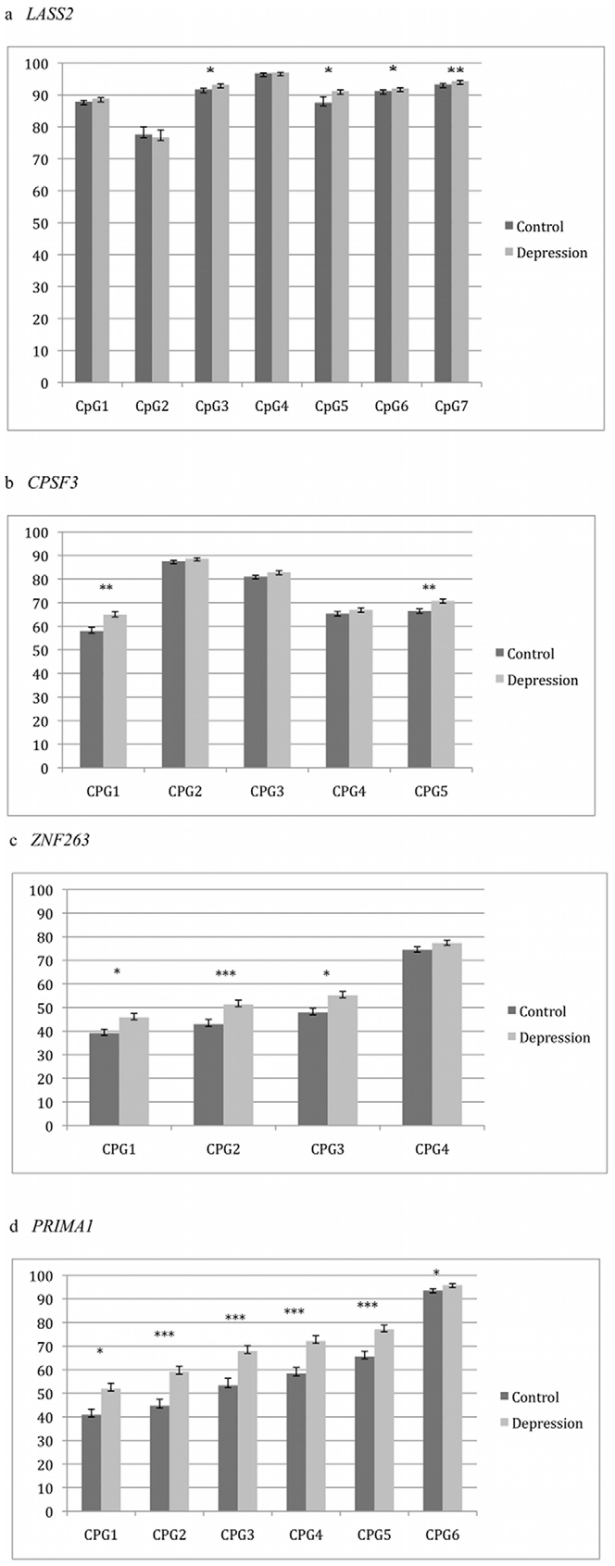
Results of bisulfite pyrosequencing for validation of CHARM in brain samples. Regions in or near four genes showed differences that remained statistically significant after correction for having tested 17 genes: (a) *LASS2*, (b) *CPSF3*, (c) *ZNF263*, (d) *PRIMA1*. The grey bars represent values from control brain sample DNA, while the black bars represent those from MDD brain samples. The Y-axis is percent DNA methylation, while the X-axis shows distance along the chromosome for each CpG dinucleotide assayed. One asterisk indicates a difference between MDD and control of p<0.05. Two asterisks indicates p<0.0029 (a correction for 17 regions tested). Three asterisks indicates p<0.00054 (a correction for 92 CpGs tested).

### Brain DNA

Postmortem frontal cortex brain tissue, Brodmann area 10, from 39 individuals with MDD and 27 matched controls were donated by the Stanley Medical Research Institute, in two batches. The first sample set consisted of 12 psychotic depression cases, 12 non-psychotic depression cases and 12 age and sex matched controls. A second set consisted of 15 non-psychotic depression cases and 15 age and sex matched controls. To increase power, the two samples were analyzed together. A structured interview-based DSM-IV diagnosis was assigned to each sample independently by two senior psychiatrists, based on available medical records and a series of interviews conducted with the family [22]. For each brain, the cerebrum was hemisected, and one half was fixed in formalin while the other was cut into 1.5 cm thick coronal slices and frozen in a mixture of isopentane and dry ice. Right and left brain hemispheres were randomly alternated for formalin fixing or freezing. Frozen tissues were used for the DNAm studies. Formalin-fixed, paraffin-embedded sections were employed for the immunohistochemical analysis of acetylcholinesterase (AChE). All frozen tissue was stored at −70°C. DNA was extracted using the MasterPure DNA Purification kit (Epicentre Biotechnologies). A replication sample set was provided by the Maryland Psychiatric Research Center. This consisted of post-mortem BA10 samples from 16 subjects with MDD and 13 controls. These samples were age, sex, and race matched.

**Table 2:** *PRIMA1* DNAm by diagnosis, and by covariate status^a.^

	MDD vs. control			Covariates (p-values)						
CpG	Control % DNAm	MDD % DNAm	Dx (p-value)	PMI	Brain pH	Side of Brain	Age	Sex	Smoking	Alcohol
1	40.3	50.9	*0.0062*	0.66	0.062	0.99	0.053	0.57	0.30	0.74
2	43.4	58.7	*0.00027*	0.50	*0.031*	0.90	*0.044*	0.88	0.51	0.71
3	51.9	67.2	*0.00028*	0.76	0.052	0.72	0.063	0.64	0.58	0.55
4	57.3	71.4	*0.00026*	0.76	0.052	0.80	0.083	0.85	0.52	0.60
5	64.4	76.7	*0.00019*	0.91	0.083	0.96	0.072	0.95	0.70	0.46
6	93.5	95.6	0.050	0.06	*0.034*	0.77	0.010	0.50	0.36	0.89

a Dx = diagnosis; PMI = postmortem interval; DNAm = DNA methylation; p-values<0.05 are italicized for clarity.

### Lymphoblastoid cell line DNA

Cases of MDD (N = 30) were selected from the Genetics of Recurrent Early Onset Depression (GenRED) study. Clinical methods have been described elsewhere [23]. MDD cases had two or more episodes of DSM-IV MDD with onset before age 31. Subjects gave written informed consent under IRB-approved protocols. European-American controls (N = 30) selected from the NIMH Genetics Initiative repository had no MDD. DNA for GenRED cases and MGS controls was provided from EBV-transformed lymphoblastoid cell lines by the NIMH Center for Collaborative Genetics Studies. A replication set of 90 MDD cases and 90 controls were also run.

**Figure 3:**
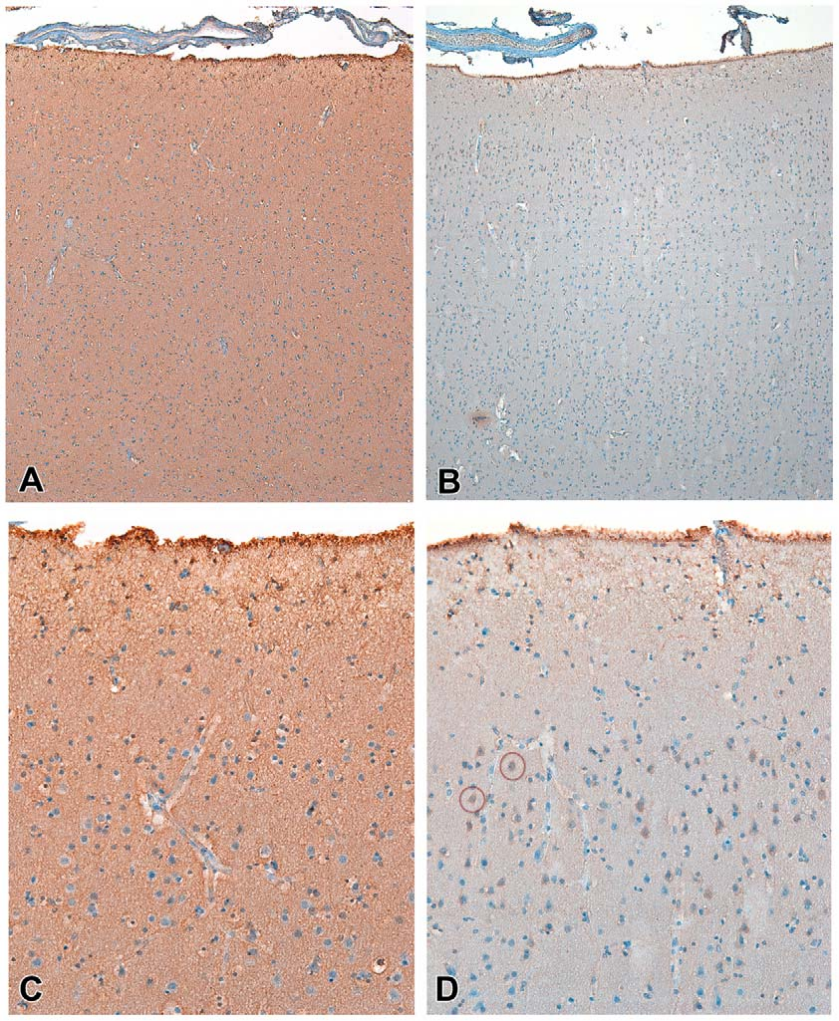
Immunohistochemical pattern of AChE in frontal cortex. (A) In controls there is diffuse and intense pattern of immunoreactivity involving mainly the neuropil. (B) In MDD subjects, though variable, immunostaining was reduced. Both 200×. (C) In controls, there is virtually no perikaryal staining. (D) The latter contrasts with the pattern observed in some areas in MDD subjects, in which groups of pyramidal neurons display intense perikaryal staining, suggesting redistribution of the enzyme to the cell body. The red circles highlight examples. Both 640×.

### CHARM platform

The CHARM assay was performed as described previously [20]. Briefly, 10 µg of DNA were sheared in 100 µl using a Hydroshear device (Genomic Solution) to 1.6 kb–3 kb. Sheared DNA was then divided into two fractions. One fraction was digested overnight at 37°C with the methyl-sensitive enzyme *Mcr*BC (NEB). Following digestion cut and uncut fractions from the same sample were electrophoresed in adjacent wells of a 1% agarose gel. Areas corresponding to the 1.6 kb–3 kb regions were excised and purified using Qiagen Spin Gel Purification columns. The gel-purified DNA was quantified on a spectrophotometer and 30 ng of DNA from each fraction was amplified using a GenomePlex Whole Genome Amplification Kit (SIGMA). The amplified DNA was then isolated with a Qiagen PCR Purification column, then quantified using a spectrophotometer. The untreated, total DNA fraction was labeled with Cy3 and the methyl-depleted DNA fraction was labeled with Cy5 and hybridized onto the custom NimbleGen 2.1 M feature CHARM microarray (design previously described [24]).

### Pyrosequencing

1 µg of genomic DNA was bisulfite treated using the Epitect kit (Qiagen). CpG unbiased primers were designed to PCR amplify 92 CpG sites in 17 genes. Nested PCR was performed. Amplicons were analyzed on a PSQ HS 96 pyrosequencer (Biotage), and CpG sites were quantified, from 0% to 100% methylation, using Pyro Q-CpG software [25].

### Real-time gene expression

RNA was extracted from frontal cortex using the RNAeasy Kit (Qiagen). MonsterScript 1st – Strand cDNA Synthesis Kit (Epicentre) was used to generate cDNA for subsequent quantitative real-time PCR. Negative RT samples were used to ensure the absence of contamination. All reactions were carried out in triplicate using 1× TaqMan master mix (Applied Biosystems), 1× TaqMan probe for each gene, and 10 ng of template in a volume of 20 µL. Real-time reactions were performed on an Applied Biosystems 7900HT Real-Time PCR System. Each set of triplicates was checked to ensure that the threshold cycle (Ct) values were all within 1 Ct of each other. The delta-delta-Ct method was used to determine sample quantity.

### Microarray data preprocessing

Hybridization quality was assessed by comparing the untreated fraction signal intensity for each genomic probe to that of background (anti-genomic) probes, with the expectation that the genomic probes should register significantly higher signals. Poor hybridization was indicated by genomic probe signal levels not being significantly higher than background probe levels. Using this metric eight arrays were identified as having failed hybridization and discarded.

### Detection of differentially methylated regions (DMRs)

Normalized methylation log-ratios were smoothed using a weighted sliding window as previously described [24]. For each probe, the average log-ratio and standard deviation were computed for cases and controls allowing a Z-score to be calculated for each probe. Under the assumption that most regions are not differentially methylated, the median absolute deviation of t-scores across all probes was used to determine the standard deviation of the null distribution. Contiguous regions of ≥6 smoothed Z-scores with p<0.005 were identified as candidate DMRs. For these regions, a Bayesian model was used to convert log ratios of intensities to estimated percent methylation [26]. P-values were assigned by comparing the DMR areas to a null distribution generated by permuting sample labels.

### Gene Ontology analysis

We sought to determine whether our nominally significant differentially methylated regions were in or near genes that clustered together functionally. We determined the nearest gene for each differential DNAm region and thus created a list of genes with differential DNAm. We then asked whether this gene list was enriched for GO Biological Process categories [27] using the NIH DAVID tool [28]. We calculated an expected number of genes we would see from our data set in each category under the null hypothesis and compared that with the observed number to obtain a p-value using the Fisher exact test. To better determine the statistical significance of these results we further calculated a False Discovery Rate using the Benjamini-Hochberg method [29].

### Analysis of pyrosequencing data

For each of the 17 most differentially methylated regions, we assessed pyrosequencing data based on primers designed across the most CpG dense part of the region implicated by CHARM. A linear regression model was used to assess the statistical significance of the effect of case-control status on DNAm. These were then corrected at two levels of stringency: 1) taking the best p-value for each gene and correcting for 17 tests (the number of regions tested); and 2) taking all p-values and correcting for 92 tests (the number of CpGs tested). We then tested DNAm levels at all CpGs against a number of additional sample variables including: pH, postmorterm interval, age, sex, side of brain assayed, smoking at time of death, and lifetime alcohol use, using a univariate regression model. Resulting p-values were corrected for the number of tests performed (92). For *PRIMA1* each of these was added as a covariate into a regression equation with case status as the primary independent variable and DNAm as the dependent variable.

### Immunohistochemistry

Ten micron-thick paraffin sections from four subjects with MDD and five controls were processed for AChE immunostaining. Sections were incubated with a rabbit polyclonal antibody targeting a signature epitope of an AChE precursor recombinant protein, particularly suitable for tissue immunohistochemistry (HPA019704; Sigma, St. Louis, MO), at a 1∶25 dilution and subsequently processed by a modification of the avidin-biotin-peroxidase method as we have previously described [30]. Several AChE immunostaining parameters were measured semi-quantitatively in a blind fashion, using Likert scale scores (0–4) as reported [30]: overall intensity of staining, degree of reticular neuropil staining, and density of perikaryal neurite clusters. Scores were compared by the Mann-Whitney-U test. In addition, we performed qualitative evaluations of neuronal perikaryal and nuclear staining.

## Results

Characteristics of postmortem brain samples are provided in Table 1. Of these 66 samples, 58 were used in our analyses. Data for eight were removed because of inadequate quality of array hybridization. CHARM analysis identified 438 nominally significant candidate DMRs between MDD and controls (Table S1). Of these, 224 DMRs showed differences >10%; the largest difference was 22%. Figure 1 shows examples of two regions with the greatest DNAm differences. We note that their magnitude was modest compared to another disease vs. control CHARM experiment in which we observed colon cancer vs. normal colon DNAm differences of up to 52%. Nonetheless, their magnitude was not unexpected given the results of a comparable study of psychiatric brain samples with DNAm differences in the single digits [19]. We calculated a false discovery rate (FDR) for each DMR to account for multiple testing. None of the DNAm differences reached the threshold for statistical significance (q-value<0.1) after correcting for multiple testing. However, we sought to further characterize the results with additional exploratory analyses.

We assessed the DNAm differences between MDD and controls using the Biological Processes categories of the Gene Ontology database [27]. The set of overrepresented categories includes many processes related to neurogenesis and central nervous system development (Table S2). These categories are intriguing given the neurotrophic model of MDD that posits a critical role for deficits in neuronal growth in the etiopathogenesis of the illness [31].

We attempted validation for 17 DMRs chosen because they were among those showing the greatest DNAm differences between MDD and controls, and were in or near genes. Within these regions bisulfite pyrosequencing was conducted across 92 CpG dinucleotides. We observed nominally significant DNAm differences in 10 of the regions. The four regions with the strongest results, those in or near the genes *LASS2*, *CPSF3*, *ZNF263*, and *PRIMA1* (Figure 2), remained statistically significant after correcting for 17 tests. The greatest DNAm difference for each gene was 4, 8, 8, and 15 percent, respectively, with the MDD samples being the more highly methylated for each of the four. When we corrected for 92 CpGs tested, only four consecutive CpGs in *PRIMA1*, with 12–15% increased DNAm in MDD, remained significant (p = 0.00019–0.00028).

For all of the 17 regions tested, we tested the impact of additional demographic, clinical, and biologic variables on DNAm (Table S3). After correction for 17 regions tested, DNAm was not predicted by pH, post-mortem interval, age, sex, side of brain, smoking, psychotic status, or alcohol use. For *PRIMA1*, two variables predicted DNAm for CpG-2 at a nominal level of significance: increased age was associated with decreased DNAm (p = 0.04), as was lower pH (p = 0.03) (Table 2). When these two variables were included as covariates in a regression the relationship between MDD and DNAm remained significant (p = 0.008–0.02). We further examined whether medication usage might account for the increased DNAm at *PRIMA1* in MDD samples by focusing on the subset of seven samples that were medication free. DNAm for these were 3–6% greater than for the remaining 32 MDD samples (p = 0.15), suggesting that medication was not responsible for the difference between MDD and controls.

To assess the potential functional impact of increased *PRIMA1* DNAm in MDD, we tested mRNA levels of the gene in the same brain samples that were used for the DNAm experiments. Levels were altered in the MDD brain samples in the expected direction, being decreased 53% (p = 0.047).

Because of the potential clinical value of blood-derived biomarkers, we sought to determine whether *PRIMA1* DNAm differences could be detected between subjects from our GenRED study as compared to normal controls collected for genetic studies. We used DNA from these subjects' lymphoblastoid cell lines and saw results similar to those in brain. DNAm was increased in MDD subjects as compared to controls (by 7–10%, p = 0.0006–0.01) for three of the four *PRIMA1* CpGs (Figure S1).

We attempted to replicate both the brain and the blood results using independent sample sets. In 16 MDD postmortem brain samples and 13 controls, we failed to detect a significant difference in DNAm at any of the four previously implicated *PRIMA1* CpGs. DNAm levels were virtually identical between groups (Supporting Information S1). The biggest difference was a 4.4% decrease in methylation for the cases at the third CpG (p = 0.16). Similarly DNAm in CpGs in *LASS2*, *CPSF3*, *ZNF263* did not differ significantly between groups (Supporting Information S1). When we examined lymphoblastoid cell line DNA from an additional 90 MDD cases and 90 controls, we could not replicate the DNAm differences in *PRIMA1* observed in the prior sample set (Supporting Information S1).

Using immunohistochemistry, we investigated whether MDD subjects with high DNAm and low expression for *PRIMA1* would show reduced immunoreactivity for AChE as compared to controls with the opposite pattern. Such a result would be consistent with the changes we observed in *PRIMA1* DNAm influencing cholinergic transmission. In a semi-quantitative comparison between frontal cortex tissues from four MDD subjects and five controls, we found that overall AChE staining intensity was reduced in the MDD subjects on average 42%, however, this difference did not reach statistical significance (p = 0.08). We also observed that subjects with MDD had a larger number of superficial pyramidal neuron perikaryal staining, despite overall reduction in neuropil immunoreactivity, suggesting redistribution of AChE towards the cell bodies (Figure 3).

## Discussion

We report here on the first genome-wide DNA methylation comparison between MDD and control brain. Although the magnitude of DNAm differences we observed was relatively small and did not survive correction for multiple testing, the DMRs identified were in or near genes enriched for roles in neuronal growth and development, suggesting that the differences picked up by our CHARM experiment, despite being relatively small, might be biologically meaningful. Our validation experiment showed the greatest differences in *PRIMA1*, with 12–15% increased DNAm in MDD. Consistent with this result, *PRIMA1* expression was decreased in MDD brain samples. The DNAm changes in the brain were also reflected in DNA from an initial set of lymphoblastoid cell lines, with MDD cases again showing greater DNAm than controls. However, we were unable to replicate *PRIMA1* DNAm differences in additional sample sets of brain and lymphoblastoid cell lines. Further, although we observed decreased immunoreactivity for AChE in MDD tissues that had increased *PRIMA1* DNAm, this change did not reach statistical significance. Therefore, we cannot draw firm conclusions about a potential role for *PRIMA1* DNAm in MDD.

*PRIMA1* is of substantial biological interest in MDD because of its relationship to cholinergic neurotransmission. The gene encodes a protein that both guides the transport of acetylcholinesterase to neuronal membranes [32] and anchors it there [33]. When *PRIMA1* is knocked down by antisense cDNA [33] or knocked out [32], there is a decrease in localization of AChE at the neuronal membrane, or of AChE activity, respectively. AChE hydrolyzes acetylcholine, thus less of its activity means more cholinergic transmission. Janowsky and colleagues proposed that increased cholinergic transmission is a central mechanism in depression, noting that reserpine, which can cause depression, is cholinomimetic, and the tricyclic antidepressants are anticholinergic [34]. Additional evidence in support of this hypothesis includes the induction of depressive symptoms by the administration of physostigmine, a more specific cholinometic agent [35], and the alleviation of such symptoms by the use of more specific anticholinergic medications such as scopolamine [36], a muscarinic acetylcholine receptor antagonist, and mecamylamine, a nicotinic acetycholine receptor antagonist [37]. Intriguingly, stress, which plays a key role in MDD etiology, has been shown to influence cholinergic gene expression in mouse brain [38].

Compared to DNAm differences seen in prior studies using the CHARM platform to compare tissue or cell types, or colon cancer vs. normal colon, the magnitude of those seen in our study was modest. This is, perhaps, not surprising given the findings of the only other genome-wide DNAm studies in psychiatric illness, that of Mill et al [19] and Dempster et al [39], which similarly found small, though statistically significant, differences between cases and controls. It is likely because the magnitude of our DNAm differences hovered around the limit of resolution of CHARM that a number of our candidate DMRs did not validate. Since completing this experiment, we have developed improvements to CHARM that increase its signal-to-noise ratio. In addition, the next generation of CHARM includes coverage of a greater number of CpGs, augmenting beyond the ∼20% of all CpGs that were initially on the array. In the current experiment we employed a conservative statistical threshold to guard against false positives. It is possible that a relaxed threshold might have captured more signals reflecting true biological differences between depression and controls.

Our failure to detect a robustly replicating signal makes it hard to draw firm conclusions about a role for DNAm in the frontal cortex of subjects with MDD. It is possible that larger etiopathologically relevant DNAm changes might exist in other brain regions known to be involved in MDD, such as the limbic regions anterior cingulate cortex [40], amygdala, and hippocampus [41]. We have previously shown brain region-specific variation in DNAm [10]. Further, disease-related DNAm variation might be restricted to particular cell types, such as neurons only or even, more narrowly, subtypes of neurons, such as pyramidal cells. However, there may be a substantial portion of DMRs that are not cell type- or tissue-specific. We note that these generalized MDD DMRs might be the most valuable as they both shed light on etiopathogenesis, and also potentially provide biomarkers that can be studied in living patients. Blood-based DMRs would also allow for much larger numbers of samples to be assayed and for correlation on a large scale with genotype.

## Supporting Information

Figure S1 **Results of bisulfite pyrosequencing of six** ***PRIMA1*** **CpGs in lymphablastoid cell line samples.** The grey bars represent values from control sample DNA, while the black bars represent those from MMD samples. The Y-axis is percent DNA methylation, while the X-axis shows each CpG arrayed along the chromosome. Asterisks indicate a difference between MDD and control of p<0.01.
(TIFF)

Table S1 **The results of the primary experiment using CHARM to compare postmortem brain samples between MDD cases and controls.**
(DOC)

Table S2 **The result of taking all genes in or near nominally significant differentially methylated regions and examining their representation in Gene Ontology Categories.**
(DOC)

Table S3 **Bisulfite pyrosequencing was used to experimentally validate some of the regions that showed differential methylation between MDD and controls by CHARM analysis.** This table shows those that were nominally validated. P-values for regression of pyrosequencing methylation at individual CpGs (rows) on 6 covariates (columns). The last column shows the F-statistic p-value for the multiple regression of methylation on all 6 covariates.
(DOC)

Supporting Information S1 **Supplementary Tables S4, S5, S6 show results of replication attempts in postmortem brain and in lymphoblastoid cell lines.**
(DOCX)
