# Supplementary figures and images for: Genome-Wide DNA Methylation Scan in Major Depressive Disorder
Source: PLoS One. 2012 Apr 12;7(4):e34451. doi: 10.1371/journal.pone.0034451 (PMC3325245; doi:10.1371/journal.pone.0034451)

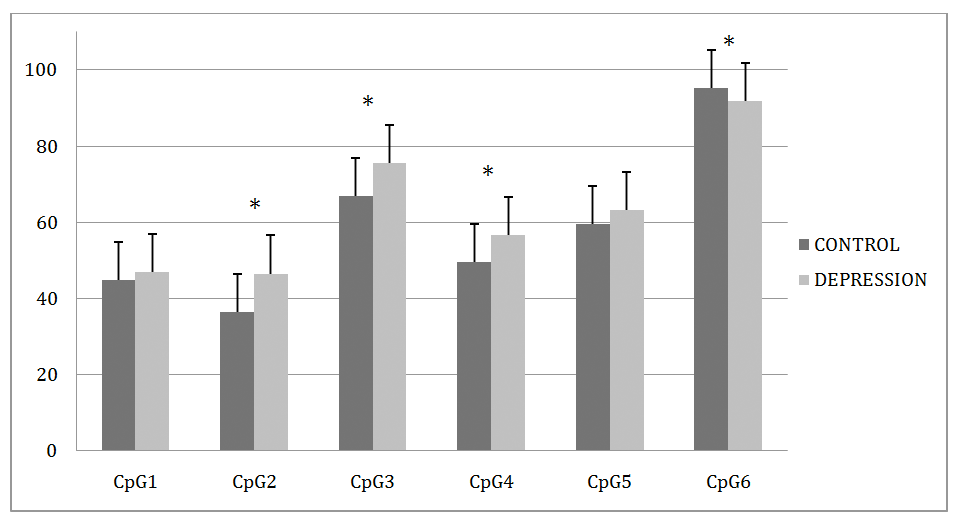

Supplement: Figure S1 — Results of bisulfite pyrosequencing of six PRIMA1 CpGs in lymphablastoid cell line samples. The grey bars represent values from control sample DNA, while the black bars represent those from MMD samples. The Y-axis is percent DNA methylation, while the X-axis shows each CpG arrayed along the chromosome. Asterisks indicate a difference between MDD and control of p<0.01. (TIFF) [file pone.0034451.s001.tiff]
