# Supplementary material for: Genome-Wide DNA Methylation Scan in Major Depressive Disorder
Source: PLoS One. 2012 Apr 12;7(4):e34451. doi: 10.1371/journal.pone.0034451 (PMC3325245; doi:10.1371/journal.pone.0034451)
Supplement: Table S1 — The results of the primary experiment using CHARM to compare postmortem brain samples between MDD cases and controls. (DOC) [file pone.0034451.s002.doc]

| **Table S1: Nominally significant candidate DMRs based on CHARM differences between MDD and controls** | | | | | | | | | | |
| --- | --- | --- | --- | --- | --- | --- | --- | --- | --- | --- |
| Chr | Start | End | DNAm % control | DNAm % MDD | DMR area | Gene | Distance to transcriptional start site (bp) | Relation to gene | P-value | Q-value |
|
| chr8 | 65,453,584 | 65,455,486 | 42.2% | 30.1% | 6.54 | BHLHB5 | 199,881 | upstream | 0.0001 | 0.3045 |
| chr14 | 93,321,105 | 93,323,210 | 49.4% | 60.8% | 5.69 | PRIMA1 | 1,308 | inside intron | 0.00017 | 0.3045 |
| chr7 | 142,690,684 | 142,692,458 | 62.8% | 73.1% | 5.13 | TMEM139 | 0 | overlaps 5' | 0.00029 | 0.3045 |
| chr9 | 73,252,369 | 73,253,461 | 52.4% | 67.8% | 4.95 | TMEM2 | 319,766 | downstream | 0.00033 | 0.3045 |
| chr10 | 105,606,436 | 105,608,476 | 69.0% | 78.7% | 4.84 | SH3PXD2A | 1,283 | promoter | 0.00037 | 0.3045 |
| chr1 | 177,258,109 | 177,260,790 | 74.7% | 83.0% | 4.82 | FAM20B | 906 | promoter | 0.00037 | 0.3045 |
| chr7 | 142,692,596 | 142,693,724 | 47.5% | 61.9% | 4.75 | TMEM139 | 412 | covers exon(s) | 0.0004 | 0.3045 |
| chr12 | 619,020 | 620,579 | 61.3% | 75.3% | 4.64 | NINJ2 | 22,436 | inside intron | 0.00044 | 0.3045 |
| chr8 | 29,172,522 | 29,173,732 | 40.1% | 53.4% | 4.39 | KIF13B | 2,827 | inside intron | 0.00056 | 0.3282 |
| chr1 | 149,205,007 | 149,206,339 | 69.4% | 80.5% | 4.32 | LASS2 | 7,724 | covers exon(s) | 0.00059 | 0.3282 |
| chr6 | 19,942,341 | 19,943,421 | 56.2% | 69.3% | 4.18 | ID4 | 2,174 | promoter | 0.00069 | 0.3400 |
| chr3 | 69,872,606 | 69,873,800 | 38.8% | 50.5% | 4.09 | MITF | 1,284 | inside intron | 0.00076 | 0.3400 |
| chr20 | 21,445,837 | 21,447,172 | 42.7% | 32.4% | 4.03 | NKX2-2 | 3,174 | upstream | 0.0008 | 0.3400 |
| chr21 | 44,230,453 | 44,232,005 | 62.0% | 70.5% | 3.84 | AGPAT3 | 60,747 | overlaps 3' | 0.00099 | 0.3905 |
| chr10 | 71,682,313 | 71,683,856 | 46.4% | 55.7% | 3.65 | NPFFR1 | 12,297 | downstream | 0.0012 | 0.4165 |
| chr7 | 150,470,645 | 150,471,734 | 53.1% | 64.6% | 3.65 | CENTG3 | 55,887 | covers exon(s) | 0.0012 | 0.4165 |
| chrX | 50,574,135 | 50,575,395 | 26.9% | 36.5% | 3.57 | SHROOM4 | 352 | promoter | 0.00133 | 0.4341 |
| chr3 | 148,620,350 | 148,621,505 | 67.5% | 57.6% | 3.35 | ZIC1 | 10,480 | downstream | 0.00173 | 0.4827 |
| chr10 | 129,976,636 | 129,977,351 | 47.3% | 63.0% | 3.32 | MKI67 | 161,992 | upstream | 0.0018 | 0.4827 |
| chr20 | 4,748,256 | 4,749,949 | 48.7% | 56.9% | 3.29 | RASSF2 | 2,341 | inside intron | 0.00188 | 0.4827 |
| chr18 | 10,717,332 | 10,718,230 | 42.9% | 58.0% | 3.17 | FAM38B | 29,519 | upstream | 0.00216 | 0.4827 |
| chr1 | 219,978,976 | 219,980,062 | 37.9% | 47.8% | 3.15 | DUSP10 | 2,021 | inside intron | 0.00223 | 0.4827 |
| chr12 | 107,571,939 | 107,575,799 | 59.8% | 69.1% | 3.14 | CORO1C | 73,624 | covers exon(s) | 0.00224 | 0.4827 |
| chr8 | 53,465,244 | 53,466,159 | 54.8% | 66.6% | 3.07 | ST18 | 18,696 | inside intron | 0.00243 | 0.4827 |
| chr15 | 29,410,412 | 29,411,795 | 43.4% | 52.5% | 3.02 | KLF13 | 4,038 | inside intron | 0.0026 | 0.4827 |
| chr15 | 41,448,049 | 41,448,715 | 72.1% | 87.1% | 2.99 | ZNF690 | 834 | covers exon(s) | 0.0027 | 0.4827 |
| chr9 | 102,276,585 | 102,278,282 | 23.9% | 30.8% | 2.95 | TMEFF1 | 1,048 | inside intron | 0.00287 | 0.4827 |
| chr7 | 36,155,998 | 36,156,580 | 38.0% | 57.7% | 2.95 | KIAA1706 | 2,780 | upstream | 0.00287 | 0.4827 |
| chr14 | 104,335,698 | 104,337,055 | 44.8% | 58.8% | 2.93 | AKT1 | 2,574 | upstream | 0.00291 | 0.4827 |
| chr18 | 42,748,350 | 42,749,636 | 28.0% | 37.7% | 2.92 | PIAS2 | 1,827 | inside intron | 0.00299 | 0.4827 |
| chr6 | 158,323,448 | 158,324,522 | 41.1% | 51.8% | 2.9 | SYNJ2 | 542 | inside intron | 0.00305 | 0.4827 |
| chr15 | 23,877,406 | 23,878,151 | 26.9% | 41.4% | 2.89 | ATP10A | 217,444 | upstream | 0.00307 | 0.4827 |
| chrX | 46,964,575 | 46,965,556 | 15.9% | 25.8% | 2.87 | PCTK1 | 1,517 | inside intron | 0.00315 | 0.4827 |
| chr8 | 120,755,024 | 120,756,305 | 30.2% | 41.7% | 2.87 | ENPP2 | 34,738 | upstream | 0.00317 | 0.4827 |
| chr17 | 78,225,872 | 78,227,500 | 67.0% | 76.2% | 2.84 | RAB40B | 22,301 | inside intron | 0.00326 | 0.4827 |
| chr1 | 45,857,754 | 45,858,954 | 53.1% | 64.4% | 2.82 | CCDC17 | 3,305 | overlaps 3' | 0.00336 | 0.4827 |
| chr14 | 101,900,640 | 101,902,335 | 28.2% | 35.9% | 2.78 | KIAA0329 | 1,510 | inside intron | 0.00354 | 0.4827 |
| chr17 | 33,859,130 | 33,859,865 | 59.8% | 72.4% | 2.77 | SOCS7 | 97,600 | downstream | 0.00358 | 0.4827 |
| chr7 | 73,343,591 | 73,345,961 | 47.1% | 54.6% | 2.76 | CYLN2 | 1,851 | inside intron | 0.00367 | 0.4827 |
| chr9 | 70,980,428 | 70,981,356 | 45.0% | 56.4% | 2.72 | TJP2 | 1,520 | inside intron | 0.00385 | 0.4827 |
| chr13 | 31,897,083 | 31,898,653 | 44.1% | 53.8% | 2.71 | CG018 | 8,244 | upstream | 0.00389 | 0.4827 |
| chr1 | 32,576,561 | 32,578,116 | 39.4% | 49.7% | 2.7 | MARCKSL1 | 2,152 | promoter | 0.00396 | 0.4827 |
| chr1 | 44,648,627 | 44,649,323 | 51.3% | 64.0% | 2.67 | C1orf164 | 5,081 | inside intron | 0.00407 | 0.4827 |
| chr1 | 207,987,725 | 207,988,719 | 45.7% | 57.8% | 2.67 | TRAF3IP3 | 7,427 | upstream | 0.00411 | 0.4827 |
| chr21 | 42,514,535 | 42,515,699 | 27.8% | 35.6% | 2.66 | ABCG1 | 1,459 | inside intron | 0.00418 | 0.4827 |
| chr10 | 22,807,699 | 22,808,894 | 31.8% | 41.3% | 2.65 | PIP5K2A | 234,595 | downstream | 0.00419 | 0.4827 |
| chr9 | 70,927,871 | 70,928,518 | 84.7% | 62.6% | 2.64 | FXN | 87,708 | downstream | 0.00424 | 0.4827 |
| chr18 | 19,338,929 | 19,341,048 | 37.9% | 51.7% | 2.62 | C18orf8 | 1,470 | covers exon(s) | 0.00435 | 0.4827 |
| chr18 | 8,777,701 | 8,778,311 | 49.8% | 65.0% | 2.59 | KIAA0802 | 70,333 | inside intron | 0.00457 | 0.4827 |
| chr1 | 35,792,819 | 35,794,207 | 23.7% | 31.3% | 2.57 | KIAA0319L | 1,383 | inside intron | 0.0047 | 0.4827 |
| chr1 | 172,393,119 | 172,393,884 | 45.9% | 57.4% | 2.55 | RABGAP1L | 1,372 | promoter | 0.00487 | 0.4827 |
| chr15 | 97,422,494 | 97,423,420 | 63.3% | 75.4% | 2.54 | DMN | 39,388 | upstream | 0.0049 | 0.4827 |
| chr16 | 30,290,036 | 30,290,981 | 38.8% | 47.9% | 2.53 | TBC1D10B | 1,635 | promoter | 0.00494 | 0.4827 |
| chr10 | 3,812,062 | 3,813,242 | 43.1% | 34.5% | 2.5 | KLF6 | 4,212 | covers exon(s) | 0.00519 | 0.4827 |
| chr3 | 130,199,907 | 130,200,812 | 41.8% | 53.6% | 2.48 | CCDC48 | 31,169 | upstream | 0.00535 | 0.4827 |
| chr6 | 163,488,765 | 163,489,515 | 53.0% | 64.1% | 2.46 | PACRG | 420,612 | inside intron | 0.00552 | 0.4827 |
| chr15 | 80,127,874 | 80,128,612 | 63.5% | 74.6% | 2.45 | RKHD3 | 2,459 | promoter | 0.0056 | 0.4827 |
| chr5 | 16,752,032 | 16,753,152 | 58.5% | 68.3% | 2.44 | MYO10 | 235,986 | covers exon(s) | 0.00564 | 0.4827 |
| chrX | 70,359,987 | 70,360,797 | 57.2% | 67.3% | 2.44 | GJB1 | 187 | overlaps exon | 0.00568 | 0.4827 |
| chr7 | 21,435,357 | 21,436,200 | 35.5% | 45.2% | 2.43 | SP4 | 1,144 | overlaps exon | 0.00573 | 0.4827 |
| chr7 | 828,041 | 828,917 | 69.1% | 59.8% | 2.43 | UNC84A | 5,264 | inside intron | 0.00573 | 0.4827 |
| chr6 | 19,916,008 | 19,916,718 | 65.5% | 78.9% | 2.42 | ID4 | 28,877 | upstream | 0.00582 | 0.4827 |
| chr16 | 85,124,672 | 85,125,599 | 39.8% | 49.7% | 2.39 | MTHFSD | 20,742 | inside intron | 0.00607 | 0.4827 |
| chr1 | 119,335,373 | 119,336,703 | 33.5% | 40.9% | 2.37 | TBX15 | 1,672 | promoter | 0.00623 | 0.4827 |
| chr10 | 2,805,304 | 2,806,105 | 57.0% | 66.8% | 2.37 | PFKP | 293,646 | upstream | 0.00627 | 0.4827 |
| chr17 | 30,437,771 | 30,438,880 | 29.7% | 39.9% | 2.35 | RFFL | 1,526 | inside intron | 0.0065 | 0.4827 |
| chr3 | 171,555,396 | 171,556,062 | 57.6% | 69.3% | 2.34 | SKIL | 2,147 | promoter | 0.0065 | 0.4827 |
| chr11 | 66,794,773 | 66,795,646 | 64.8% | 55.9% | 2.33 | ADRBK1 | 4,105 | inside intron | 0.00661 | 0.4827 |
| chrX | 128,895,488 | 128,896,257 | 29.6% | 39.7% | 2.33 | UTP14A | 27,649 | downstream | 0.00668 | 0.4827 |
| chr8 | 65,445,931 | 65,446,705 | 59.8% | 49.7% | 2.32 | BHLHB5 | 208,662 | upstream | 0.00673 | 0.4827 |
| chr11 | 74,916,208 | 74,917,728 | 77.4% | 83.6% | 2.32 | GDPD5 | 1,694 | promoter | 0.00679 | 0.4827 |
| chr11 | 2,645,926 | 2,647,180 | 80.0% | 86.3% | 2.3 | KCNQ1 | 206,667 | inside intron | 0.00694 | 0.4827 |
| chr16 | 86,314,062 | 86,314,695 | 43.6% | 55.6% | 2.29 | KLHDC4 | 42,347 | inside intron | 0.00703 | 0.4827 |
| chr1 | 84,238,621 | 84,239,428 | 28.9% | 38.4% | 2.28 | TTLL7 | 1,201 | promoter | 0.00715 | 0.4827 |
| chr2 | 47,989,223 | 47,989,925 | 68.0% | 78.8% | 2.27 | FBXO11 | 2,906 | upstream | 0.00725 | 0.4827 |
| chr8 | 123,864,945 | 123,865,925 | 30.9% | 40.8% | 2.27 | ZHX2 | 1,864 | inside intron | 0.00731 | 0.4827 |
| chr17 | 59,578,844 | 59,579,540 | 36.6% | 47.4% | 2.26 | TEX2 | 114,844 | inside exon | 0.00734 | 0.4827 |
| chr3 | 135,608,761 | 135,609,463 | 35.2% | 46.0% | 2.26 | AMOTL2 | 32,666 | upstream | 0.00737 | 0.4827 |
| chr17 | 76,339,520 | 76,340,255 | 41.8% | 52.1% | 2.26 | KIAA1303 | 206,301 | inside intron | 0.00741 | 0.4827 |
| chr1 | 152,793,949 | 152,794,829 | 64.6% | 73.3% | 2.25 | UBE2Q1 | 2,914 | covers exon(s) | 0.00747 | 0.4827 |
| chr11 | 2,218,192 | 2,218,921 | 49.3% | 63.3% | 2.25 | ASCL2 | 29,836 | downstream | 0.00754 | 0.4827 |
| chr6 | 36,752,488 | 36,753,996 | 30.1% | 36.7% | 2.24 | CDKN1A | 468 | promoter | 0.00757 | 0.4827 |
| chr2 | 46,009,351 | 46,010,053 | 61.0% | 50.4% | 2.23 | PRKCE | 276,805 | inside intron | 0.0077 | 0.4827 |
| chr5 | 177,946,841 | 177,947,435 | 40.4% | 53.6% | 2.23 | COL23A1 | 2,726 | inside intron | 0.00773 | 0.4827 |
| chr8 | 141,605,827 | 141,606,780 | 71.7% | 79.6% | 2.23 | EIF2C2 | 108,046 | downstream | 0.00777 | 0.4827 |
| chr10 | 122,695,631 | 122,696,297 | 38.0% | 49.1% | 2.22 | BRWD2 | 94,947 | downstream | 0.0079 | 0.4827 |
| chr10 | 14,687,808 | 14,688,297 | 44.2% | 58.9% | 2.21 | FAM107B | 168,604 | inside intron | 0.00798 | 0.4827 |
| chr13 | 100,118,544 | 100,119,306 | 51.7% | 63.3% | 2.2 | TMTC4 | 26,057 | upstream | 0.00804 | 0.4827 |
| chr11 | 66,949,624 | 66,951,976 | 40.5% | 55.1% | 2.2 | RPS6KB2 | 534 | promoter | 0.00813 | 0.4827 |
| chr15 | 65,916,323 | 65,916,887 | 53.1% | 66.0% | 2.19 | LBXCOR1 | 11,329 | downstream | 0.00816 | 0.4827 |
| chr3 | 51,970,941 | 51,971,660 | 60.3% | 72.5% | 2.19 | PCBP4 | 287 | inside intron | 0.00825 | 0.4827 |
| chr9 | 36,128,999 | 36,129,711 | 61.3% | 72.2% | 2.19 | C9orf19 | 2,258 | inside intron | 0.00826 | 0.4827 |
| chr4 | 185,625,307 | 185,626,114 | 46.5% | 55.5% | 2.17 | IRF2 | 6,582 | inside intron | 0.0085 | 0.4827 |
| chr3 | 99,025,472 | 99,026,177 | 35.7% | 46.0% | 2.17 | ARL6 | 59,188 | downstream | 0.00851 | 0.4827 |
| chr10 | 626,950 | 628,238 | 73.3% | 81.0% | 2.16 | DIP2C | 97,367 | inside intron | 0.00864 | 0.4827 |
| chr20 | 2,615,014 | 2,616,897 | 68.5% | 76.8% | 2.15 | IDH3B | 22,172 | upstream | 0.00869 | 0.4827 |
| chr10 | 22,661,349 | 22,662,154 | 62.1% | 71.1% | 2.15 | BMI1 | 11,204 | downstream | 0.0087 | 0.4827 |
| chr7 | 139,121,120 | 139,122,203 | 23.6% | 30.4% | 2.15 | TBXAS1 | 53,217 | upstream | 0.00876 | 0.4827 |
| chr6 | 43,349,758 | 43,350,421 | 35.7% | 46.4% | 2.14 | TTBK1 | 30,559 | inside intron | 0.00892 | 0.4827 |
| chr17 | 33,862,552 | 33,863,074 | 35.4% | 48.7% | 2.14 | SOCS7 | 101,022 | downstream | 0.00896 | 0.4827 |
| chr16 | 15,654,429 | 15,654,990 | 52.8% | 65.3% | 2.13 | NDE1 | 2,825 | inside intron | 0.00902 | 0.4827 |
| chrX | 11,684,431 | 11,685,210 | 31.2% | 43.0% | 2.12 | MSL3L1 | 988 | promoter | 0.00911 | 0.4827 |
| chr2 | 46,763,953 | 46,764,580 | 31.2% | 42.3% | 2.11 | SOCS5 | 15,022 | upstream | 0.00927 | 0.4827 |
| chr4 | 141,394,232 | 141,395,189 | 29.5% | 38.6% | 2.1 | SCOC | 2,700 | upstream | 0.00944 | 0.4827 |
| chr11 | 2,672,543 | 2,673,629 | 70.3% | 77.3% | 2.1 | KCNQ1 | 233,284 | inside intron | 0.00947 | 0.4827 |
| chr14 | 75,917,641 | 75,918,421 | 49.4% | 58.5% | 2.1 | ESRRB | 10,163 | inside intron | 0.00949 | 0.4827 |
| chr11 | 3,816,810 | 3,817,856 | 32.9% | 40.9% | 2.09 | RHOG | 903 | inside intron | 0.00957 | 0.4827 |
| chr10 | 6,663,596 | 6,664,194 | 42.5% | 55.5% | 2.09 | PRKCQ | 1,353 | promoter | 0.00957 | 0.4827 |
| chr15 | 45,794,021 | 45,795,021 | 40.0% | 49.5% | 2.09 | SEMA6D | 2,956 | upstream | 0.0096 | 0.4827 |
| chr1 | 211,253,144 | 211,253,633 | 56.6% | 70.4% | 2.08 | ANGEL2 | 2,157 | overlaps exon | 0.00971 | 0.4827 |
| chr1 | 42,275,900 | 42,276,353 | 50.9% | 65.8% | 2.08 | GUCA2B | 115,325 | upstream | 0.00974 | 0.4827 |
| chrX | 47,307,571 | 47,309,028 | 56.8% | 69.0% | 2.07 | ARAF | 2,121 | overlaps exon | 0.00982 | 0.4827 |
| chr14 | 22,901,038 | 22,901,599 | 34.3% | 46.5% | 2.07 | EFS | 3,082 | inside intron | 0.00984 | 0.4827 |
| chr19 | 16,060,922 | 16,061,372 | 40.6% | 55.3% | 2.06 | TPM4 | 12,598 | overlaps exon | 0.01002 | 0.4876 |
| chr19 | 44,570,944 | 44,572,032 | 75.9% | 82.3% | 2.04 | PAF1 | 1,486 | covers exon(s) | 0.01034 | 0.4984 |
| chr11 | 2,865,050 | 2,865,645 | 74.0% | 81.8% | 2.03 | SLC22A18AS | 15,900 | downstream | 0.01061 | 0.5041 |
| chr12 | 50,075,104 | 50,075,999 | 58.8% | 68.0% | 2.02 | GALNT6 | 3,638 | upstream | 0.0107 | 0.5041 |
| chr6 | 170,416,948 | 170,417,605 | 61.4% | 72.5% | 2 | DLL1 | 24,016 | downstream | 0.01102 | 0.5041 |
| chr5 | 139,464,621 | 139,465,464 | 23.8% | 31.8% | 2 | PURA | 8,427 | upstream | 0.01105 | 0.5041 |
| chr20 | 61,043,558 | 61,044,359 | 43.3% | 56.6% | 2 | C20orf11 | 3,673 | inside intron | 0.01114 | 0.5041 |
| chr13 | 111,755,139 | 111,755,805 | 62.1% | 52.2% | 1.98 | SOX1 | 14,108 | upstream | 0.01139 | 0.5041 |
| chr2 | 130,828,204 | 130,828,765 | 44.4% | 56.0% | 1.98 | PTPN18 | 1,322 | promoter | 0.01142 | 0.5041 |
| chr2 | 86,871,904 | 86,872,929 | 60.9% | 68.5% | 1.98 | CD8A | 267 | promoter | 0.01144 | 0.5041 |
| chr11 | 29,979,060 | 29,979,619 | 54.4% | 66.0% | 1.97 | KCNA4 | 15,444 | downstream | 0.01156 | 0.5041 |
| chr19 | 52,436,987 | 52,437,569 | 34.7% | 46.3% | 1.97 | BBC3 | 10,697 | upstream | 0.01166 | 0.5041 |
| chr11 | 2,661,032 | 2,661,878 | 68.7% | 76.8% | 1.96 | KCNQ1 | 221,773 | inside intron | 0.01183 | 0.5041 |
| chr5 | 128,456,705 | 128,457,402 | 37.5% | 51.5% | 1.96 | ISOC1 | 938 | promoter | 0.01183 | 0.5041 |
| chr7 | 42,245,184 | 42,245,898 | 60.6% | 70.0% | 1.96 | GLI3 | 15,765 | upstream | 0.01183 | 0.5041 |
| chr4 | 185,634,055 | 185,635,192 | 46.2% | 56.5% | 1.95 | IRF2 | 1,359 | promoter | 0.01189 | 0.5041 |
| chr11 | 74,822,002 | 74,822,636 | 57.6% | 69.0% | 1.95 | GDPD5 | 91,878 | downstream | 0.01193 | 0.5041 |
| chr9 | 98,419,097 | 98,420,054 | 49.1% | 57.9% | 1.94 | CDC14B | 1,878 | inside intron | 0.01215 | 0.5041 |
| chr1 | 191,356,259 | 191,356,941 | 28.5% | 38.2% | 1.94 | CDC73 | 842 | promoter | 0.01218 | 0.5041 |
| chr11 | 2,322,153 | 2,322,816 | 65.7% | 75.4% | 1.93 | TSPAN32 | 41,640 | downstream | 0.01229 | 0.5041 |
| chr17 | 69,708,369 | 69,709,044 | 59.9% | 69.6% | 1.93 | RPL38 | 2,345 | promoter | 0.01237 | 0.5041 |
| chr5 | 139,262,376 | 139,262,973 | 69.1% | 79.8% | 1.92 | NRG2 | 140,089 | inside intron | 0.01253 | 0.5041 |
| chr9 | 132,698,485 | 132,699,079 | 51.2% | 61.8% | 1.92 | ABL1 | 119,397 | inside intron | 0.01263 | 0.5041 |
| chr3 | 148,605,448 | 148,606,048 | 69.6% | 58.4% | 1.91 | ZIC4 | 1,048 | inside intron | 0.01271 | 0.5041 |
| chr8 | 1,748,475 | 1,749,141 | 79.8% | 89.4% | 1.91 | ARHGEF10 | 10,414 | upstream | 0.01285 | 0.5041 |
| chr19 | 50,235,063 | 50,235,660 | 53.7% | 64.2% | 1.9 | SFRS16 | 926 | covers exon(s) | 0.01294 | 0.5041 |
| chr12 | 33,484,593 | 33,485,566 | 63.8% | 72.4% | 1.9 | SYT10 | 573 | promoter | 0.01298 | 0.5041 |
| chr8 | 42,025,137 | 42,025,710 | 65.3% | 76.5% | 1.89 | MYST3 | 2,924 | inside exon | 0.01312 | 0.5041 |
| chr4 | 24,639,860 | 24,640,556 | 58.1% | 67.1% | 1.89 | LGI2 | 856 | inside intron | 0.01314 | 0.5041 |
| chrX | 50,571,983 | 50,572,712 | 25.5% | 34.9% | 1.88 | SHROOM4 | 1,071 | inside intron | 0.01338 | 0.5041 |
| chr1 | 247,070,866 | 247,071,446 | 69.0% | 80.0% | 1.88 | SH3BP5L | 15,330 | overlaps 3' | 0.01344 | 0.5041 |
| chr9 | 23,813,224 | 23,813,861 | 46.6% | 36.7% | 1.88 | ELAVL2 | 2,201 | inside intron | 0.01352 | 0.5041 |
| chr6 | 138,228,200 | 138,228,846 | 42.2% | 53.9% | 1.87 | TNFAIP3 | 1,427 | promoter | 0.01361 | 0.5041 |
| chr10 | 42,289,748 | 42,290,198 | 49.4% | 62.7% | 1.87 | ZNF33B | 163,799 | downstream | 0.01369 | 0.5041 |
| chr18 | 74,836,378 | 74,836,939 | 44.9% | 33.9% | 1.87 | SALL3 | 4,323 | upstream | 0.01371 | 0.5041 |
| chrX | 106,903,532 | 106,904,306 | 27.9% | 38.3% | 1.86 | TSC22D3 | 1,366 | inside intron | 0.0138 | 0.5041 |
| chr11 | 2,679,558 | 2,680,470 | 65.4% | 72.3% | 1.86 | KCNQ1 | 240,299 | inside intron | 0.01383 | 0.5041 |
| chr6 | 112,516,541 | 112,517,126 | 22.0% | 32.9% | 1.86 | LOC619208 | 1,175 | inside intron | 0.01393 | 0.5041 |
| chr1 | 202,033,851 | 202,034,376 | 49.9% | 61.5% | 1.86 | ZC3H11A | 2,478 | inside intron | 0.01393 | 0.5041 |
| chr8 | 22,071,909 | 22,072,885 | 56.7% | 66.5% | 1.85 | LGI3 | 1,621 | promoter | 0.014 | 0.5041 |
| chr15 | 28,985,371 | 28,985,824 | 45.5% | 58.8% | 1.85 | KIAA1018 | 1,951 | overlaps exon | 0.01407 | 0.5041 |
| chr20 | 17,538,065 | 17,538,724 | 46.4% | 57.3% | 1.85 | DSTN | 39,467 | downstream | 0.01409 | 0.5041 |
| chr12 | 109,894,175 | 109,894,703 | 62.8% | 74.3% | 1.84 | MYL2 | 51,412 | upstream | 0.0143 | 0.5080 |
| chr7 | 126,676,947 | 126,677,574 | 25.6% | 35.3% | 1.84 | GRM8 | 6,402 | upstream | 0.01438 | 0.5080 |
| chr17 | 61,726,798 | 61,727,625 | 69.1% | 77.9% | 1.83 | PRKCA | 1,762 | promoter | 0.01456 | 0.5112 |
| chr3 | 99,722,460 | 99,722,910 | 39.6% | 52.6% | 1.82 | CLDND1 | 1,689 | overlaps exon | 0.01477 | 0.5137 |
| chr7 | 131,913,630 | 131,914,296 | 73.3% | 82.4% | 1.82 | PLXNA4B | 69,690 | inside intron | 0.01482 | 0.5137 |
| chr19 | 17,049,018 | 17,049,615 | 48.3% | 58.4% | 1.81 | NY-SAR-48 | 1,676 | promoter | 0.01504 | 0.5181 |
| chr6 | 167,233,288 | 167,235,943 | 91.9% | 94.4% | 1.8 | RNASET2 | 54,123 | downstream | 0.01532 | 0.5229 |
| chr12 | 131,467,604 | 131,469,380 | 14.1% | 22.7% | 1.8 | GALNT9 | 211,079 | upstream | 0.01537 | 0.5229 |
| chr19 | 5,159,694 | 5,160,045 | 39.0% | 55.2% | 1.78 | PTPRS | 131,768 | inside intron | 0.01581 | 0.5343 |
| chr1 | 14,092,683 | 14,093,487 | 61.5% | 53.8% | 1.78 | PRDM2 | 188,747 | downstream | 0.01591 | 0.5343 |
| chr10 | 131,823,502 | 131,824,024 | 27.5% | 38.6% | 1.77 | TXNL2 | 628 | promoter | 0.01604 | 0.5343 |
| chr11 | 31,781,392 | 31,782,061 | 48.4% | 39.1% | 1.77 | PAX6 | 7,372 | inside intron | 0.01617 | 0.5343 |
| chr1 | 145,112,513 | 145,113,047 | 34.2% | 45.3% | 1.76 | PRKAB2 | 1,761 | promoter | 0.01625 | 0.5343 |
| chr7 | 92,075,312 | 92,075,862 | 39.4% | 53.0% | 1.76 | CDK6 | 225,285 | inside exon | 0.01628 | 0.5343 |
| chr11 | 1,862,154 | 1,862,643 | 61.2% | 72.9% | 1.75 | LSP1 | 13,425 | covers exon(s) | 0.0165 | 0.5358 |
| chr6 | 132,765,401 | 132,766,103 | 70.6% | 78.9% | 1.75 | MOXD1 | 1,077 | promoter | 0.01652 | 0.5358 |
| chr3 | 12,982,604 | 12,983,450 | 36.6% | 43.6% | 1.75 | IQSEC1 | 509 | inside intron | 0.0167 | 0.5386 |
| chr15 | 59,310,800 | 59,311,342 | 56.9% | 67.7% | 1.73 | RORA | 2,007 | promoter | 0.017 | 0.5401 |
| chr1 | 7,691,580 | 7,692,296 | 62.6% | 71.3% | 1.73 | CAMTA1 | 923,610 | inside intron | 0.01703 | 0.5401 |
| chr12 | 123,148,590 | 123,149,359 | 59.4% | 68.5% | 1.73 | ZNF664 | 124,968 | downstream | 0.0171 | 0.5401 |
| chr11 | 22,320,415 | 22,321,043 | 38.5% | 29.4% | 1.73 | SLC17A6 | 4,173 | inside intron | 0.01718 | 0.5401 |
| chr10 | 690,154 | 690,715 | 42.3% | 52.4% | 1.73 | DIP2C | 34,890 | inside intron | 0.01724 | 0.5401 |
| chr13 | 36,394,785 | 36,395,223 | 37.1% | 50.4% | 1.72 | ALG5 | 76,253 | downstream | 0.01734 | 0.5402 |
| chr20 | 33,048,988 | 33,049,480 | 25.9% | 37.3% | 1.72 | MYH7B | 22,122 | overlaps exon | 0.01746 | 0.5409 |
| chr17 | 25,728,563 | 25,728,970 | 33.2% | 50.3% | 1.7 | CPD | 1,139 | promoter | 0.01789 | 0.5513 |
| chr8 | 28,978,248 | 28,978,842 | 63.8% | 73.8% | 1.7 | KIF13B | 197,717 | downstream | 0.01811 | 0.5549 |
| chr1 | 154,447,793 | 154,448,249 | 54.5% | 66.6% | 1.69 | SLC25A44 | 17,271 | inside exon | 0.01822 | 0.5551 |
| chr2 | 71,359,018 | 71,359,548 | 22.7% | 33.3% | 1.69 | ZNF638 | 52,848 | upstream | 0.01833 | 0.5553 |
| chr8 | 76,481,141 | 76,481,810 | 33.2% | 24.3% | 1.69 | HNF4G | 132,947 | upstream | 0.01844 | 0.5557 |
| chr17 | 34,085,806 | 34,086,423 | 40.0% | 50.5% | 1.68 | MLLT6 | 28,977 | upstream | 0.01854 | 0.5557 |
| chr14 | 67,068,435 | 67,068,888 | 48.4% | 60.4% | 1.67 | PLEKHH1 | 872 | promoter | 0.01883 | 0.5615 |
| chr2 | 66,523,935 | 66,525,018 | 82.4% | 77.0% | 1.67 | MEIS1 | 7,900 | inside intron | 0.01903 | 0.5644 |
| chr1 | 167,340,539 | 167,340,992 | 42.1% | 54.0% | 1.66 | ATP1B1 | 1,578 | promoter | 0.01935 | 0.5696 |
| chrX | 52,969,337 | 52,969,931 | 58.1% | 67.3% | 1.65 | TMEM29 | 24,751 | downstream | 0.01949 | 0.5696 |
| chr11 | 35,642,174 | 35,642,570 | 46.6% | 64.9% | 1.65 | TRIM44 | 1,246 | inside intron | 0.01971 | 0.5696 |
| chr5 | 83,052,323 | 83,053,021 | 33.0% | 42.7% | 1.65 | HAPLN1 | 0 | overlaps 5' | 0.01972 | 0.5696 |
| chr20 | 9,768,566 | 9,769,124 | 20.1% | 29.7% | 1.65 | PAK7 | 1,045 | promoter | 0.01973 | 0.5696 |
| chr2 | 98,592,768 | 98,593,329 | 67.0% | 76.7% | 1.64 | UNC50 | 1,295 | overlaps exon | 0.01982 | 0.5696 |
| chr8 | 1,956,780 | 1,957,200 | 41.4% | 53.9% | 1.63 | KBTBD11 | 47,330 | downstream | 0.0203 | 0.5747 |
| chr16 | 961,560 | 962,216 | 58.7% | 68.9% | 1.63 | TMEM112 | 576 | promoter | 0.02044 | 0.5747 |
| chr12 | 105,502,704 | 105,503,403 | 41.1% | 33.3% | 1.63 | RFX4 | 1,542 | inside intron | 0.02047 | 0.5747 |
| chr5 | 16,236,531 | 16,237,056 | 69.3% | 79.5% | 1.62 | FBXL7 | 683,227 | downstream | 0.02051 | 0.5747 |
| chr5 | 179,212,218 | 179,212,572 | 56.9% | 71.6% | 1.62 | LOC51149 | 5,873 | inside intron | 0.02052 | 0.5747 |
| chr11 | 109,482,228 | 109,482,717 | 55.8% | 66.6% | 1.62 | RDX | 189,929 | downstream | 0.02065 | 0.5753 |
| chr14 | 94,853,971 | 94,854,670 | 17.0% | 24.7% | 1.62 | CLMN | 1,284 | inside intron | 0.02077 | 0.5758 |
| chr10 | 73,440,858 | 73,441,521 | 67.3% | 75.3% | 1.61 | CHST3 | 46,733 | inside exon | 0.02092 | 0.5773 |
| chr3 | 66,635,547 | 66,636,058 | 43.5% | 55.0% | 1.61 | LRIG1 | 2,013 | promoter | 0.02108 | 0.5786 |
| chr10 | 102,758,080 | 102,758,536 | 36.7% | 48.1% | 1.61 | LZTS2 | 11,126 | downstream | 0.02121 | 0.5795 |
| chr1 | 35,213,393 | 35,214,022 | 55.7% | 66.3% | 1.59 | ZMYM6 | 56,133 | downstream | 0.02167 | 0.5880 |
| chr9 | 80,043,813 | 80,044,407 | 27.3% | 36.2% | 1.59 | PSAT1 | 57,471 | upstream | 0.02181 | 0.5880 |
| chr6 | 90,175,301 | 90,175,721 | 40.9% | 53.2% | 1.59 | RRAGD | 2,762 | inside intron | 0.02186 | 0.5880 |
| chr9 | 138,557,262 | 138,557,928 | 45.9% | 55.2% | 1.58 | NOTCH1 | 2,130 | inside intron | 0.02222 | 0.5880 |
| chr7 | 43,763,958 | 43,764,237 | 47.9% | 65.4% | 1.58 | BLVRA | 559 | promoter | 0.02223 | 0.5880 |
| chr20 | 4,750,021 | 4,750,582 | 47.1% | 56.4% | 1.58 | RASSF2 | 1,708 | inside intron | 0.02225 | 0.5880 |
| chr22 | 28,441,898 | 28,442,713 | 42.4% | 52.9% | 1.58 | CABP7 | 3,630 | upstream | 0.02226 | 0.5880 |
| chr1 | 210,655,364 | 210,655,889 | 33.8% | 43.6% | 1.58 | C1orf75 | 505 | promoter | 0.02239 | 0.5881 |
| chr3 | 55,499,993 | 55,500,915 | 76.2% | 82.8% | 1.57 | WNT5A | 3,623 | upstream | 0.0226 | 0.5881 |
| chr13 | 35,440,567 | 35,441,050 | 45.2% | 56.4% | 1.57 | DCAMKL1 | 162,392 | inside intron | 0.02275 | 0.5881 |
| chr15 | 83,003,531 | 83,003,948 | 33.2% | 45.3% | 1.57 | NMB | 726 | promoter | 0.02276 | 0.5881 |
| chr7 | 6,582,296 | 6,582,791 | 72.3% | 82.7% | 1.56 | ZDHHC4 | 1,875 | promoter | 0.02317 | 0.5881 |
| chr21 | 35,089,820 | 35,090,273 | 49.3% | 60.4% | 1.55 | RUNX1 | 92,583 | inside intron | 0.02323 | 0.5881 |
| chr2 | 235,527,999 | 235,528,701 | 46.6% | 54.0% | 1.55 | SH3BP4 | 2,633 | inside intron | 0.02326 | 0.5881 |
| chr12 | 32,148,806 | 32,149,656 | 56.4% | 67.5% | 1.55 | BICD1 | 1,795 | promoter | 0.02341 | 0.5881 |
| chr1 | 32,027,868 | 32,028,412 | 43.5% | 53.2% | 1.55 | SPOCD1 | 25,754 | downstream | 0.02342 | 0.5881 |
| chr12 | 121,923,097 | 121,924,462 | 52.0% | 61.6% | 1.55 | VPS37B | 22,202 | inside intron | 0.02352 | 0.5881 |
| chr2 | 17,924,981 | 17,925,480 | 38.4% | 49.4% | 1.55 | KCNS3 | 1,556 | inside intron | 0.02363 | 0.5881 |
| chr17 | 40,746,938 | 40,747,391 | 43.7% | 54.7% | 1.54 | MAP3K14 | 2,805 | inside intron | 0.02364 | 0.5881 |
| chr11 | 82,119,652 | 82,120,666 | 14.6% | 19.7% | 1.53 | MGC33846 | 1,887 | close to 3' | 0.02408 | 0.5881 |
| chr1 | 212,236,443 | 212,236,932 | 45.0% | 55.2% | 1.53 | PROX1 | 7,961 | inside exon | 0.02414 | 0.5881 |
| chr4 | 1,159,532 | 1,160,054 | 68.3% | 77.8% | 1.53 | SPON2 | 2,936 | upstream | 0.02422 | 0.5881 |
| chr16 | 45,472,485 | 45,474,134 | 78.4% | 83.8% | 1.53 | GPT2 | 1,674 | promoter | 0.02425 | 0.5881 |
| chr1 | 22,137,629 | 22,138,298 | 56.1% | 63.8% | 1.53 | HSPG2 | 1,296 | promoter | 0.02428 | 0.5881 |
| chr11 | 133,792,794 | 133,793,214 | 47.9% | 59.6% | 1.53 | B3GAT1 | 5,773 | upstream | 0.0244 | 0.5881 |
| chr19 | 43,436,464 | 43,438,349 | 26.5% | 34.6% | 1.52 | PPP1R14A | 662 | inside intron | 0.02454 | 0.5881 |
| chr18 | 11,973,808 | 11,974,402 | 68.9% | 77.3% | 1.52 | IMPA2 | 2,354 | inside intron | 0.02478 | 0.5881 |
| chr1 | 243,146,760 | 243,147,387 | 69.2% | 77.2% | 1.52 | HNRPU | 52,311 | upstream | 0.02481 | 0.5881 |
| chrX | 100,069,470 | 100,069,995 | 41.5% | 50.9% | 1.52 | XKRX | 558 | overlaps exon | 0.02488 | 0.5881 |
| chr14 | 73,252,268 | 73,252,640 | 49.8% | 63.5% | 1.51 | C14orf43 | 44,113 | inside exon | 0.02491 | 0.5881 |
| chr14 | 68,511,619 | 68,512,735 | 58.2% | 49.3% | 1.51 | ACTN1 | 2,973 | inside intron | 0.02501 | 0.5881 |
| chr2 | 47,024,449 | 47,024,902 | 44.2% | 55.0% | 1.51 | TTC7A | 2,633 | inside intron | 0.02507 | 0.5881 |
| chr10 | 79,463,278 | 79,463,800 | 32.9% | 23.5% | 1.51 | RPS24 | 0 | overlaps 5' | 0.02515 | 0.5881 |
| chr9 | 99,782,505 | 99,782,928 | 34.0% | 45.6% | 1.51 | ANP32B | 2,535 | upstream | 0.02522 | 0.5881 |
| chr19 | 48,838,595 | 48,839,365 | 71.2% | 80.6% | 1.5 | PLAUR | 26,976 | downstream | 0.0254 | 0.5881 |
| chr15 | 21,445,513 | 21,446,661 | 41.9% | 54.4% | 1.5 | NDN | 36,881 | downstream | 0.02546 | 0.5881 |
| chr9 | 122,677,525 | 122,677,927 | 40.6% | 54.2% | 1.5 | PHF19 | 1,499 | inside intron | 0.02569 | 0.5881 |
| chr7 | 75,797,700 | 75,798,120 | 40.8% | 52.3% | 1.5 | YWHAG | 28,131 | inside intron | 0.02582 | 0.5881 |
| chr17 | 44,654,557 | 44,655,010 | 65.4% | 76.0% | 1.49 | ABI3 | 11,970 | overlaps exon | 0.02613 | 0.5881 |
| chr6 | 53,518,447 | 53,518,765 | 45.9% | 60.7% | 1.49 | GCLC | 658 | promoter | 0.02622 | 0.5881 |
| chr17 | 41,333,295 | 41,334,478 | 21.8% | 27.3% | 1.49 | MAPT | 5,672 | inside intron | 0.02628 | 0.5881 |
| chr3 | 19,252,623 | 19,252,938 | 46.6% | 61.4% | 1.48 | KCNH8 | 87,603 | inside intron | 0.02633 | 0.5881 |
| chr11 | 2,636,388 | 2,636,919 | 54.2% | 64.1% | 1.48 | KCNQ1 | 197,129 | inside intron | 0.02634 | 0.5881 |
| chr5 | 43,053,447 | 43,054,047 | 47.8% | 39.5% | 1.48 | LOC389289 | 22,050 | downstream | 0.02642 | 0.5881 |
| chr14 | 20,560,748 | 20,561,726 | 25.5% | 30.6% | 1.48 | NDRG2 | 1,377 | covers exon(s) | 0.02645 | 0.5881 |
| chr20 | 19,688,869 | 19,689,109 | 42.8% | 61.3% | 1.48 | SLC24A3 | 547,580 | downstream | 0.02649 | 0.5881 |
| chr3 | 124,226,674 | 124,227,223 | 31.6% | 43.0% | 1.48 | SEMA5B | 2,042 | inside intron | 0.02651 | 0.5881 |
| chr2 | 9,482,592 | 9,483,232 | 43.0% | 59.4% | 1.48 | CPSF3 | 1,274 | inside intron | 0.02662 | 0.5881 |
| chr14 | 103,265,204 | 103,265,834 | 70.3% | 62.6% | 1.47 | ZFYVE21 | 13,307 | overlaps exon | 0.02686 | 0.5912 |
| chr19 | 12,957,028 | 12,957,586 | 22.8% | 31.4% | 1.47 | NFIX | 9,997 | upstream | 0.02711 | 0.5924 |
| chr1 | 201,201,166 | 201,201,829 | 66.5% | 76.2% | 1.46 | CYB5R1 | 1,197 | covers exon(s) | 0.02744 | 0.5924 |
| chr13 | 78,082,079 | 78,082,535 | 57.5% | 67.9% | 1.46 | C13orf7 | 48,779 | downstream | 0.02759 | 0.5924 |
| chr15 | 99,845,180 | 99,845,942 | 75.2% | 82.5% | 1.46 | PCSK6 | 1,767 | inside intron | 0.02769 | 0.5924 |
| chr11 | 2,223,565 | 2,224,177 | 42.2% | 50.7% | 1.46 | ASCL2 | 24,580 | downstream | 0.02778 | 0.5924 |
| chr16 | 65,983,308 | 65,983,731 | 35.8% | 47.0% | 1.45 | CGI-38 | 1,190 | inside intron | 0.02808 | 0.5924 |
| chr12 | 123,560,211 | 123,560,771 | 35.9% | 44.4% | 1.45 | NCOR2 | 25,330 | inside intron | 0.02809 | 0.5924 |
| chr8 | 31,008,436 | 31,008,859 | 57.5% | 68.7% | 1.45 | PURG | 999 | inside exon | 0.02823 | 0.5924 |
| chr15 | 29,409,677 | 29,410,133 | 32.8% | 43.2% | 1.45 | KLF13 | 3,303 | inside intron | 0.02829 | 0.5924 |
| chr17 | 54,643,829 | 54,644,302 | 32.1% | 42.4% | 1.44 | C17orf71 | 1,677 | overlaps exon | 0.02844 | 0.5924 |
| chr1 | 80,279,265 | 80,279,757 | 38.8% | 48.4% | 1.44 | IFI44 | 1,391,162 | downstream | 0.02851 | 0.5924 |
| chr5 | 27,154,316 | 27,154,815 | 47.2% | 61.5% | 1.43 | CDH9 | 79,871 | upstream | 0.0289 | 0.5924 |
| chr16 | 85,655,789 | 85,656,537 | 72.2% | 65.3% | 1.43 | FBXO31 | 318,326 | downstream | 0.02897 | 0.5924 |
| chr1 | 53,457,074 | 53,457,749 | 40.1% | 53.1% | 1.43 | C1orf123 | 1,127 | covers exon(s) | 0.02898 | 0.5924 |
| chr5 | 113,722,849 | 113,723,362 | 35.4% | 46.4% | 1.43 | KCNN2 | 2,552 | upstream | 0.02909 | 0.5924 |
| chr7 | 127,790,228 | 127,790,883 | 72.1% | 81.0% | 1.43 | RBM28 | 19,031 | upstream | 0.02915 | 0.5924 |
| chr7 | 77,483,693 | 77,484,113 | 38.7% | 49.7% | 1.43 | MAGI2 | 1,436,712 | downstream | 0.02927 | 0.5924 |
| chr6 | 33,155,034 | 33,155,544 | 36.9% | 47.1% | 1.43 | HLA-DPB1 | 3,297 | inside intron | 0.02939 | 0.5924 |
| chr1 | 59,050,928 | 59,051,448 | 29.1% | 40.1% | 1.42 | JUN | 28,556 | upstream | 0.02949 | 0.5924 |
| chr9 | 93,749,405 | 93,749,756 | 50.8% | 63.7% | 1.42 | ROR2 | 2,508 | inside intron | 0.02981 | 0.5924 |
| chr20 | 35,778,656 | 35,779,045 | 44.2% | 57.1% | 1.42 | CTNNBL1 | 22,809 | inside intron | 0.03003 | 0.5924 |
| chr11 | 1,721,356 | 1,721,773 | 57.0% | 67.9% | 1.41 | HCCA2 | 20,303 | inside intron | 0.03006 | 0.5924 |
| chr19 | 10,393,630 | 10,394,224 | 34.0% | 41.8% | 1.41 | CDC37 | 18,360 | upstream | 0.0302 | 0.5924 |
| chr12 | 56,225,879 | 56,226,266 | 46.6% | 58.3% | 1.41 | DCTN2 | 978 | covers exon(s) | 0.03026 | 0.5924 |
| chr3 | 66,001,692 | 66,002,353 | 33.8% | 42.6% | 1.41 | MAGI1 | 2,144 | promoter | 0.03034 | 0.5924 |
| chr15 | 97,701,340 | 97,701,865 | 71.4% | 80.2% | 1.41 | LRRC28 | 92,166 | inside intron | 0.0304 | 0.5924 |
| chr11 | 2,201,056 | 2,201,515 | 39.1% | 49.1% | 1.41 | ASCL2 | 47,242 | downstream | 0.03044 | 0.5924 |
| chr9 | 19,779,358 | 19,779,984 | 18.7% | 26.1% | 1.4 | SLC24A2 | 2,433 | promoter | 0.03069 | 0.5924 |
| chr7 | 100,261,379 | 100,262,188 | 38.0% | 43.9% | 1.4 | EPHB4 | 890 | inside intron | 0.03089 | 0.5924 |
| chr1 | 88,698,592 | 88,699,288 | 47.9% | 57.9% | 1.4 | PKN2 | 223,221 | upstream | 0.0309 | 0.5924 |
| chr10 | 126,671,127 | 126,671,651 | 51.2% | 61.2% | 1.4 | CTBP2 | 34,791 | covers exon(s) | 0.03096 | 0.5924 |
| chr10 | 22,655,295 | 22,655,832 | 26.4% | 35.1% | 1.4 | BMI1 | 5,150 | covers exon(s) | 0.03102 | 0.5924 |
| chr9 | 111,298,094 | 111,299,000 | 69.3% | 80.0% | 1.4 | PTPN3 | 32,540 | upstream | 0.03108 | 0.5924 |
| chr10 | 102,024,401 | 102,024,644 | 37.0% | 54.4% | 1.39 | BLOC1S2 | 11,459 | inside exon | 0.03131 | 0.5924 |
| chr12 | 121,889,578 | 121,890,043 | 45.4% | 55.3% | 1.39 | HIP1R | 3,587 | inside intron | 0.03133 | 0.5924 |
| chr20 | 56,895,915 | 56,896,338 | 62.5% | 73.2% | 1.39 | GNAS | 34,485 | inside intron | 0.03138 | 0.5924 |
| chr5 | 180,553,114 | 180,553,522 | 31.1% | 42.7% | 1.39 | TRIM7 | 7,013 | downstream | 0.0314 | 0.5924 |
| chr20 | 60,769,029 | 60,769,455 | 48.7% | 59.3% | 1.39 | SLCO4A1 | 24,788 | inside intron | 0.03158 | 0.5924 |
| chr11 | 7,231,545 | 7,232,313 | 24.1% | 30.1% | 1.39 | SYT9 | 1,789 | inside intron | 0.03158 | 0.5924 |
| chr13 | 42,886,712 | 42,887,027 | 48.3% | 62.2% | 1.39 | PIG38 | 252,627 | inside intron | 0.03159 | 0.5924 |
| chr14 | 70,356,717 | 70,357,035 | 63.8% | 77.7% | 1.39 | MAP3K9 | 11,077 | upstream | 0.03163 | 0.5924 |
| chr21 | 46,689,071 | 46,689,557 | 58.4% | 67.6% | 1.39 | PCNT | 120,589 | overlaps exon | 0.03176 | 0.5924 |
| chr12 | 26,384,170 | 26,384,554 | 62.1% | 73.6% | 1.39 | ITPR2 | 492,843 | covers exon(s) | 0.03177 | 0.5924 |
| chr17 | 59,926,784 | 59,927,204 | 35.4% | 46.0% | 1.38 | DDX5 | 5,664 | overlaps exon | 0.03189 | 0.5924 |
| chr9 | 127,313,908 | 127,314,619 | 51.6% | 60.9% | 1.38 | MAPKAP1 | 194,714 | inside intron | 0.03206 | 0.5924 |
| chr3 | 53,853,727 | 53,854,282 | 52.1% | 60.2% | 1.38 | CHDH | 933 | inside intron | 0.0321 | 0.5924 |
| chr3 | 53,051,950 | 53,052,437 | 39.2% | 49.1% | 1.38 | SFMBT1 | 1,883 | covers exon(s) | 0.03211 | 0.5924 |
| chr13 | 100,119,693 | 100,120,870 | 84.0% | 89.5% | 1.38 | TMTC4 | 27,206 | upstream | 0.03228 | 0.5924 |
| chr11 | 61,090,575 | 61,090,959 | 53.7% | 65.1% | 1.38 | SYT7 | 13,914 | inside intron | 0.0323 | 0.5924 |
| chr17 | 73,732,164 | 73,732,734 | 30.4% | 38.5% | 1.38 | BIRC5 | 10,293 | inside exon | 0.03232 | 0.5924 |
| chr19 | 4,917,068 | 4,919,390 | 50.2% | 58.8% | 1.38 | JMJD2B | 741 | promoter | 0.03237 | 0.5924 |
| chr6 | 27,172,198 | 27,172,546 | 33.1% | 45.5% | 1.37 | HIST1H2BJ | 36,007 | downstream | 0.0327 | 0.5966 |
| chr5 | 34,041,122 | 34,041,434 | 39.2% | 52.8% | 1.37 | AMACR | 2,528 | inside intron | 0.03295 | 0.5974 |
| chr12 | 50,070,656 | 50,071,106 | 34.8% | 44.5% | 1.36 | GALNT6 | 360 | covers exon(s) | 0.03314 | 0.5974 |
| chr17 | 76,651,631 | 76,652,153 | 70.2% | 61.7% | 1.36 | BAIAP2 | 28,075 | inside intron | 0.03329 | 0.5974 |
| chr14 | 59,625,050 | 59,625,658 | 49.6% | 61.9% | 1.36 | C14orf135 | 26,752 | upstream | 0.03338 | 0.5974 |
| chr7 | 150,340,481 | 150,340,871 | 49.4% | 60.7% | 1.36 | ATG9B | 11,647 | inside exon | 0.03359 | 0.5974 |
| chr7 | 149,167,551 | 149,167,971 | 61.1% | 71.5% | 1.35 | LOC401431 | 33,842 | downstream | 0.03377 | 0.5974 |
| chr2 | 238,863,150 | 238,863,687 | 29.3% | 37.7% | 1.35 | PER2 | 1,320 | promoter | 0.03385 | 0.5974 |
| chr17 | 43,138,042 | 43,138,582 | 64.4% | 72.9% | 1.35 | TBKBP1 | 10,414 | inside intron | 0.03386 | 0.5974 |
| chr7 | 150,046,674 | 150,047,305 | 60.4% | 69.4% | 1.35 | GIMAP1 | 2,044 | covers exon(s) | 0.03389 | 0.5974 |
| chr3 | 160,003,889 | 160,004,138 | 34.4% | 51.3% | 1.35 | MFSD1 | 1,284 | inside intron | 0.03408 | 0.5974 |
| chr2 | 171,385,344 | 171,385,985 | 55.7% | 46.0% | 1.35 | GAD1 | 3,899 | inside intron | 0.03414 | 0.5974 |
| chr14 | 88,955,335 | 88,955,860 | 68.3% | 76.7% | 1.35 | CHES1 | 2,209 | promoter | 0.03416 | 0.5974 |
| chr17 | 23,998,026 | 23,999,576 | 57.9% | 67.5% | 1.35 | SDF2 | 13,483 | downstream | 0.03424 | 0.5974 |
| chr15 | 81,114,930 | 81,115,212 | 53.1% | 68.1% | 1.34 | AP3B2 | 60,476 | downstream | 0.03431 | 0.5974 |
| chr16 | 4,616,265 | 4,616,763 | 57.2% | 69.4% | 1.34 | MGRN1 | 1,412 | inside intron | 0.03436 | 0.5974 |
| chr16 | 85,126,206 | 85,126,626 | 37.5% | 47.9% | 1.34 | MTHFSD | 19,715 | inside intron | 0.03447 | 0.5974 |
| chr18 | 58,532,537 | 58,533,188 | 25.7% | 32.8% | 1.34 | PHLPP | 525 | promoter | 0.03469 | 0.5993 |
| chr17 | 78,388,462 | 78,388,990 | 55.0% | 63.9% | 1.33 | ZNF750 | 2,229 | inside intron | 0.03511 | 0.6011 |
| chr8 | 145,906,695 | 145,907,325 | 73.1% | 80.1% | 1.33 | ZNF34 | 69,143 | downstream | 0.03531 | 0.6011 |
| chr6 | 4,718,114 | 4,718,744 | 19.9% | 26.8% | 1.33 | CDYL | 66,723 | inside intron | 0.03555 | 0.6011 |
| chr1 | 119,964,936 | 119,965,321 | 49.3% | 60.3% | 1.32 | PHGDH | 90,711 | upstream | 0.03571 | 0.6011 |
| chr6 | 144,370,245 | 144,370,676 | 36.9% | 26.7% | 1.32 | PLAGL1 | 569 | inside intron | 0.03576 | 0.6011 |
| chr4 | 108,858,341 | 108,859,334 | 11.9% | 16.8% | 1.32 | PAPSS1 | 1,533 | inside intron | 0.03577 | 0.6011 |
| chr2 | 242,099,657 | 242,100,479 | 71.6% | 78.9% | 1.32 | STK25 | 2,951 | upstream | 0.03578 | 0.6011 |
| chr12 | 6,747,954 | 6,748,512 | 42.0% | 34.3% | 1.32 | PTMS | 2,153 | inside intron | 0.03583 | 0.6011 |
| chr7 | 971,039 | 971,626 | 54.6% | 42.6% | 1.32 | COX19 | 10,134 | inside exon | 0.03595 | 0.6011 |
| chr18 | 51,322,989 | 51,323,370 | 35.4% | 46.4% | 1.32 | TCF4 | 83,070 | inside intron | 0.03595 | 0.6011 |
| chr10 | 124,755,583 | 124,756,537 | 17.3% | 22.4% | 1.32 | IKZF5 | 1,766 | overlaps exon | 0.03608 | 0.6011 |
| chr2 | 197,164,044 | 197,164,656 | 27.0% | 34.3% | 1.32 | HECW2 | 923 | inside intron | 0.0361 | 0.6011 |
| chr22 | 30,066,620 | 30,067,178 | 36.2% | 43.9% | 1.32 | PATZ1 | 5,070 | overlaps 3' | 0.03628 | 0.6023 |
| chr12 | 54,336,799 | 54,337,530 | 56.3% | 69.4% | 1.31 | OR10P1 | 19,857 | downstream | 0.03649 | 0.6023 |
| chr11 | 2,865,086 | 2,865,434 | 76.1% | 84.3% | 1.31 | SLC22A18AS | 16,111 | downstream | 0.03658 | 0.6023 |
| chr11 | 2,632,330 | 2,633,032 | 63.5% | 70.0% | 1.31 | KCNQ1 | 193,071 | inside intron | 0.03667 | 0.6023 |
| chrX | 152,584,105 | 152,584,540 | 59.9% | 71.8% | 1.31 | PNCK | 8,433 | downstream | 0.03671 | 0.6023 |
| chr18 | 44,639,056 | 44,639,479 | 36.8% | 47.6% | 1.3 | KIAA0427 | 319,632 | inside intron | 0.03765 | 0.6145 |
| chr17 | 65,674,453 | 65,674,854 | 45.4% | 58.4% | 1.3 | KCNJ2 | 2,416 | promoter | 0.03767 | 0.6145 |
| chr18 | 27,928,686 | 27,929,028 | 44.3% | 57.3% | 1.29 | RNF138 | 2,042 | inside intron | 0.03784 | 0.6145 |
| chr5 | 98,295,379 | 98,295,790 | 36.2% | 47.0% | 1.29 | CHD1 | 5,242 | upstream | 0.03802 | 0.6145 |
| chr18 | 43,311,196 | 43,311,580 | 35.1% | 45.9% | 1.29 | FUSSEL18 | 281,645 | upstream | 0.03814 | 0.6145 |
| chr15 | 99,849,943 | 99,850,399 | 40.8% | 50.0% | 1.29 | PCSK6 | 2,234 | promoter | 0.03828 | 0.6145 |
| chr8 | 67,616,005 | 67,616,488 | 48.2% | 57.4% | 1.29 | C8orf46 | 47,685 | downstream | 0.03837 | 0.6145 |
| chr4 | 2,252,730 | 2,253,666 | 69.2% | 77.3% | 1.29 | ZFYVE28 | 136,500 | inside intron | 0.03846 | 0.6145 |
| chr9 | 34,372,813 | 34,373,167 | 46.0% | 57.7% | 1.28 | C9orf24 | 14,681 | overlaps exon | 0.03857 | 0.6145 |
| chr18 | 9,696,547 | 9,696,931 | 64.7% | 75.4% | 1.28 | RAB31 | 1,371 | promoter | 0.03872 | 0.6145 |
| chr17 | 32,383,137 | 32,383,554 | 68.4% | 78.3% | 1.28 | AATF | 2,850 | inside intron | 0.03897 | 0.6145 |
| chr1 | 241,705,740 | 241,706,511 | 85.0% | 90.6% | 1.28 | SDCCAG8 | 219,798 | inside intron | 0.03899 | 0.6145 |
| chr12 | 50,073,479 | 50,073,935 | 78.9% | 88.0% | 1.28 | GALNT6 | 2,013 | promoter | 0.03902 | 0.6145 |
| chr11 | 2,328,857 | 2,329,355 | 71.7% | 80.8% | 1.28 | CD81 | 25,767 | upstream | 0.03913 | 0.6145 |
| chr2 | 28,644,193 | 28,645,000 | 85.7% | 91.0% | 1.28 | PLB1 | 71,708 | inside intron | 0.03921 | 0.6145 |
| chr1 | 182,274,051 | 182,274,746 | 40.1% | 51.7% | 1.28 | GLT25D2 | 566 | promoter | 0.03927 | 0.6145 |
| chr15 | 58,071,912 | 58,072,330 | 63.8% | 73.6% | 1.27 | FOXB1 | 11,382 | upstream | 0.03934 | 0.6145 |
| chr1 | 117,467,607 | 117,468,742 | 85.3% | 90.6% | 1.27 | TRIM45 | 1,674 | promoter | 0.03951 | 0.6155 |
| chr1 | 224,134,397 | 224,134,922 | 42.7% | 50.6% | 1.27 | TMEM63A | 1,748 | inside intron | 0.03964 | 0.6156 |
| chr6 | 32,836,105 | 32,836,447 | 57.0% | 69.7% | 1.27 | HLA-DQA2 | 18,965 | downstream | 0.03974 | 0.6156 |
| chr12 | 70,242,879 | 70,243,467 | 63.7% | 71.1% | 1.27 | LGR5 | 122,800 | inside intron | 0.03986 | 0.6157 |
| chr3 | 13,497,569 | 13,497,920 | 35.8% | 47.3% | 1.26 | HDAC11 | 746 | covers exon(s) | 0.04024 | 0.6173 |
| chr11 | 108,800,122 | 108,800,746 | 52.8% | 43.1% | 1.26 | LOC399947 | 2,038 | inside exon | 0.04027 | 0.6173 |
| chr19 | 40,321,917 | 40,322,326 | 61.2% | 49.7% | 1.26 | FXYD1 | 346 | inside intron | 0.0403 | 0.6173 |
| chrX | 107,864,284 | 107,864,671 | 22.7% | 33.2% | 1.26 | IRS4 | 1,623 | inside exon | 0.04059 | 0.6183 |
| chrX | 74,062,790 | 74,063,207 | 28.9% | 38.6% | 1.26 | KIAA2022 | 1,082 | promoter | 0.04062 | 0.6183 |
| chr16 | 3,276,196 | 3,276,478 | 48.8% | 62.8% | 1.26 | ZNF263 | 2,709 | inside intron | 0.0407 | 0.6183 |
| chr1 | 231,008,798 | 231,009,287 | 24.9% | 33.3% | 1.25 | C1orf57 | 143,705 | upstream | 0.04126 | 0.6214 |
| chr3 | 48,447,176 | 48,447,524 | 52.7% | 64.1% | 1.25 | CCDC51 | 9,008 | downstream | 0.04133 | 0.6214 |
| chr17 | 76,624,369 | 76,624,792 | 42.0% | 32.5% | 1.24 | BAIAP2 | 813 | inside intron | 0.04192 | 0.6214 |
| chr18 | 70,317,485 | 70,318,080 | 21.7% | 28.6% | 1.24 | CNDP2 | 2,909 | inside intron | 0.0421 | 0.6214 |
| chr13 | 73,048,386 | 73,048,875 | 79.6% | 87.9% | 1.24 | KLF12 | 557,191 | downstream | 0.0421 | 0.6214 |
| chr4 | 83,898,889 | 83,899,240 | 35.7% | 46.9% | 1.24 | SCD5 | 39,708 | inside intron | 0.04222 | 0.6214 |
| chr17 | 53,952,641 | 53,953,109 | 28.2% | 37.7% | 1.24 | 4-Sep | 8,552 | overlaps exon | 0.04233 | 0.6214 |
| chr14 | 75,914,376 | 75,914,800 | 54.0% | 63.5% | 1.24 | ESRRB | 6,898 | inside intron | 0.04238 | 0.6214 |
| chr12 | 100,978,894 | 100,979,416 | 39.6% | 47.3% | 1.24 | CCDC53 | 560 | covers exon(s) | 0.04241 | 0.6214 |
| chr2 | 28,825,772 | 28,826,225 | 25.2% | 34.0% | 1.24 | PPP1CB | 1,892 | promoter | 0.04254 | 0.6214 |
| chr11 | 2,256,837 | 2,257,125 | 56.9% | 70.6% | 1.24 | ASCL2 | 8,080 | upstream | 0.04254 | 0.6214 |
| chr1 | 7,756,360 | 7,756,750 | 43.4% | 53.7% | 1.23 | VAMP3 | 2,445 | inside intron | 0.04316 | 0.6214 |
| chr5 | 60,492,377 | 60,492,758 | 30.3% | 41.5% | 1.23 | DKFZP686E2158 | 1,290 | overlaps exon | 0.04319 | 0.6214 |
| chr9 | 964,294 | 964,808 | 42.3% | 34.1% | 1.23 | DMRT3 | 2,155 | promoter | 0.04326 | 0.6214 |
| chr6 | 146,099,774 | 146,100,159 | 33.0% | 43.2% | 1.22 | EPM2A | 1,091 | promoter | 0.04372 | 0.6214 |
| chr14 | 89,236,540 | 89,236,888 | 69.0% | 80.1% | 1.22 | C14orf143 | 253,953 | downstream | 0.04376 | 0.6214 |
| chr12 | 12,738,945 | 12,739,470 | 76.3% | 83.9% | 1.22 | CDKN1B | 22,105 | upstream | 0.0438 | 0.6214 |
| chr14 | 64,854,435 | 64,854,960 | 73.2% | 81.3% | 1.22 | FUT8 | 92,632 | upstream | 0.04383 | 0.6214 |
| chr22 | 42,588,174 | 42,588,558 | 60.3% | 50.1% | 1.22 | SULT4A1 | 1,152 | inside intron | 0.04398 | 0.6214 |
| chr2 | 70,637,563 | 70,637,956 | 44.3% | 54.4% | 1.22 | TGFA | 3,126 | upstream | 0.04399 | 0.6214 |
| chr15 | 99,835,980 | 99,836,385 | 53.4% | 63.5% | 1.22 | PCSK6 | 11,324 | inside intron | 0.044 | 0.6214 |
| chr8 | 135,796,635 | 135,797,120 | 59.1% | 69.3% | 1.22 | ZFAT1 | 2,173 | promoter | 0.04404 | 0.6214 |
| chr22 | 30,668,429 | 30,668,708 | 46.5% | 60.1% | 1.22 | YWHAH | 1,770 | promoter | 0.04416 | 0.6214 |
| chr6 | 170,385,281 | 170,385,913 | 61.2% | 70.6% | 1.22 | DLL1 | 55,708 | downstream | 0.04417 | 0.6214 |
| chr19 | 35,630,139 | 35,630,586 | 53.4% | 62.8% | 1.22 | ZNF536 | 74,972 | inside intron | 0.04417 | 0.6214 |
| chr5 | 75,736,549 | 75,736,969 | 66.0% | 75.3% | 1.22 | IQGAP2 | 1,645 | inside intron | 0.04424 | 0.6214 |
| chr17 | 59,578,142 | 59,578,565 | 24.3% | 33.6% | 1.21 | TEX2 | 115,819 | overlaps 3' | 0.04442 | 0.6214 |
| chr13 | 113,828,675 | 113,829,062 | 69.6% | 79.8% | 1.21 | RASA3 | 87,134 | inside intron | 0.0445 | 0.6214 |
| chr17 | 39,387,633 | 39,388,817 | 66.0% | 71.8% | 1.21 | PYY | 48,545 | inside intron | 0.04465 | 0.6214 |
| chr11 | 2,046,352 | 2,047,072 | 63.1% | 69.9% | 1.21 | IGF2 | 69,707 | downstream | 0.04469 | 0.6214 |
| chr5 | 135,215,219 | 135,215,929 | 63.7% | 71.3% | 1.21 | LOC153328 | 16,956 | inside intron | 0.04472 | 0.6214 |
| chr14 | 77,149,159 | 77,149,986 | 21.9% | 27.9% | 1.21 | SPTLC2 | 2,876 | inside intron | 0.04477 | 0.6214 |
| chr16 | 10,181,977 | 10,182,256 | 54.7% | 41.3% | 1.21 | GRIN2A | 1,855 | inside intron | 0.04512 | 0.6214 |
| chr5 | 72,783,845 | 72,784,641 | 63.1% | 75.1% | 1.2 | FOXD1 | 3,738 | upstream | 0.0455 | 0.6214 |
| chr9 | 94,713,834 | 94,714,596 | 46.3% | 51.8% | 1.2 | ZNF484 | 33,724 | upstream | 0.04555 | 0.6214 |
| chr11 | 719,650 | 719,971 | 49.6% | 62.9% | 1.2 | EPS8L2 | 23,528 | downstream | 0.04559 | 0.6214 |
| chr16 | 65,767,648 | 65,768,020 | 47.4% | 60.8% | 1.2 | LOC653319 | 7,363 | inside exon | 0.04567 | 0.6214 |
| chr20 | 17,612,002 | 17,612,314 | 45.1% | 57.1% | 1.2 | RRBP1 | 1,075 | promoter | 0.0457 | 0.6214 |
| chr21 | 44,389,772 | 44,390,090 | 64.4% | 52.4% | 1.2 | C21orf33 | 11,849 | overlaps 3' | 0.04575 | 0.6214 |
| chr12 | 128,903,074 | 128,903,665 | 73.1% | 79.7% | 1.2 | TMEM132D | 50,500 | inside intron | 0.04582 | 0.6214 |
| chr19 | 56,558,848 | 56,559,409 | 68.6% | 75.6% | 1.2 | ETFB | 2,074 | inside intron | 0.04585 | 0.6214 |
| chr5 | 137,051,999 | 137,052,574 | 46.5% | 57.4% | 1.2 | KLHL3 | 46,787 | inside intron | 0.04593 | 0.6214 |
| chr11 | 41,280,961 | 41,284,863 | 36.3% | 45.5% | 1.2 | LRRC4C | 1,008,722 | upstream | 0.04605 | 0.6214 |
| chr15 | 21,484,541 | 21,484,790 | 60.7% | 75.6% | 1.19 | NDN | 999 | promoter | 0.04633 | 0.6214 |
| chr11 | 61,485,889 | 61,486,588 | 74.4% | 80.1% | 1.19 | BEST1 | 11,482 | overlaps exon | 0.04642 | 0.6214 |
| chr13 | 29,939,016 | 29,939,400 | 26.2% | 36.1% | 1.19 | HMGB1 | 1,004 | promoter | 0.04664 | 0.6214 |
| chr5 | 179,427,260 | 179,427,890 | 21.8% | 28.1% | 1.19 | RNF130 | 3,824 | inside intron | 0.04667 | 0.6214 |
| chr18 | 59,140,750 | 59,141,260 | 26.5% | 34.5% | 1.19 | BCL2 | 3,158 | upstream | 0.04682 | 0.6214 |
| chr11 | 118,523,744 | 118,524,128 | 47.4% | 57.3% | 1.19 | ABCG4 | 831 | promoter | 0.04686 | 0.6214 |
| chr3 | 123,386,426 | 123,386,903 | 59.8% | 68.3% | 1.19 | CASR | 1,207 | inside intron | 0.04687 | 0.6214 |
| chr22 | 36,708,570 | 36,708,918 | 32.0% | 42.8% | 1.19 | SOX10 | 1,566 | inside intron | 0.04693 | 0.6214 |
| chr10 | 35,693,046 | 35,693,361 | 35.3% | 47.2% | 1.19 | C10orf9 | 27,086 | inside intron | 0.04711 | 0.6214 |
| chr17 | 35,028,949 | 35,029,264 | 38.3% | 50.1% | 1.19 | PPP1R1B | 7,440 | upstream | 0.04719 | 0.6214 |
| chr2 | 46,776,679 | 46,777,168 | 65.5% | 73.4% | 1.19 | SOCS5 | 2,434 | promoter | 0.04721 | 0.6214 |
| chr2 | 168,809,732 | 168,810,448 | 42.6% | 51.1% | 1.18 | STK39 | 1,902 | inside intron | 0.04744 | 0.6214 |
| chr5 | 76,365,335 | 76,365,683 | 46.2% | 56.9% | 1.18 | AGGF1 | 3,348 | inside intron | 0.04748 | 0.6214 |
| chr5 | 132,136,051 | 132,136,630 | 47.7% | 56.1% | 1.18 | FLJ16793 | 25,016 | downstream | 0.0475 | 0.6214 |
| chr2 | 202,026,656 | 202,027,142 | 27.7% | 35.6% | 1.18 | ALS2CR2 | 2,020 | inside intron | 0.04755 | 0.6214 |
| chr15 | 32,836,115 | 32,836,520 | 61.8% | 71.7% | 1.18 | CX36 | 2,135 | promoter | 0.04763 | 0.6214 |
| chr3 | 109,293,646 | 109,293,997 | 49.3% | 60.0% | 1.18 | CD47 | 1,022 | promoter | 0.04792 | 0.6214 |
| chr9 | 96,530,154 | 96,530,661 | 48.6% | 57.7% | 1.17 | C9orf3 | 1,340 | inside intron | 0.04828 | 0.6214 |
| chr4 | 54,664,100 | 54,664,580 | 42.1% | 33.1% | 1.17 | GSH2 | 3,146 | downstream | 0.04842 | 0.6214 |
| chr2 | 24,866,640 | 24,867,703 | 76.7% | 82.0% | 1.17 | LOC391356 | 2,051 | overlaps 3' | 0.04848 | 0.6214 |
| chr13 | 24,674,991 | 24,675,759 | 81.3% | 86.3% | 1.17 | FAM123A | 31,135 | upstream | 0.0485 | 0.6214 |
| chr10 | 119,290,373 | 119,290,898 | 46.6% | 38.8% | 1.17 | EMX2 | 1,047 | promoter | 0.04851 | 0.6214 |
| chr7 | 150,300,570 | 150,301,068 | 57.5% | 68.2% | 1.17 | KCNH2 | 4,878 | inside intron | 0.04853 | 0.6214 |
| chr1 | 229,179,675 | 229,179,918 | 44.2% | 58.8% | 1.17 | TTC13 | 1,288 | inside intron | 0.04853 | 0.6214 |
| chr18 | 46,338,194 | 46,338,512 | 37.8% | 49.5% | 1.17 | MAPK4 | 1,969 | promoter | 0.04863 | 0.6214 |
| chr12 | 126,195,013 | 126,195,458 | 45.4% | 62.1% | 1.17 | TMEM132B | 1,817,899 | downstream | 0.04871 | 0.6214 |
| chr4 | 155,881,767 | 155,882,148 | 73.0% | 63.3% | 1.17 | LRAT | 2,464 | promoter | 0.04874 | 0.6214 |
| chr5 | 72,784,995 | 72,785,415 | 46.9% | 55.8% | 1.17 | FOXD1 | 4,888 | upstream | 0.04919 | 0.6257 |
| chr9 | 21,323,675 | 21,324,131 | 35.2% | 43.5% | 1.16 | KLHL9 | 1,297 | inside exon | 0.0494 | 0.6269 |
| chr15 | 49,421,855 | 49,422,278 | 46.3% | 55.3% | 1.16 | GLDN | 851 | inside intron | 0.04955 | 0.6273 |
