# Supplementary material for: Genome-Wide DNA Methylation Scan in Major Depressive Disorder
Source: PLoS One. 2012 Apr 12;7(4):e34451. doi: 10.1371/journal.pone.0034451 (PMC3325245; doi:10.1371/journal.pone.0034451)
Supplement: Table S2 — The result of taking all genes in or near nominally significant differentially methylated regions and examining their representation in Gene Ontology Categories. (DOC) [file pone.0034451.s003.doc]

| **Table S2: Gene Ontology overrepresented categories following assessment of DNAm differences between MDD and controls** | | | |
| --- | --- | --- | --- |
| Term | Fold Enrichment | P-value | FDR % |
| GO:0007275~multicellular organismal development | 1.35 | 6.19E-15 | 1.16E-11 |
| GO:0048731~system development | 1.40 | 8.20E-15 | 1.53E-11 |
| GO:0007399~nervous system development | 1.59 | 1.93E-14 | 3.60E-11 |
| GO:0032502~developmental process | 1.32 | 4.69E-14 | 8.76E-11 |
| GO:0048856~anatomical structure development | 1.35 | 3.45E-13 | 6.44E-10 |
| GO:0032501~multicellular organismal process | 1.26 | 1.35E-12 | 2.51E-09 |
| GO:0022008~neurogenesis | 1.67 | 2.11E-10 | 3.94E-07 |
| GO:0048699~generation of neurons | 1.69 | 3.35E-10 | 6.25E-07 |
| GO:0007417~central nervous system development | 1.81 | 4.32E-10 | 8.07E-07 |
| GO:0048869~cellular developmental process | 1.38 | 6.97E-10 | 1.30E-06 |
| GO:0009653~anatomical structure morphogenesis | 1.46 | 9.47E-10 | 1.77E-06 |
| GO:0048513~organ development | 1.37 | 1.75E-09 | 3.27E-06 |
| GO:0030154~cell differentiation | 1.37 | 3.19E-09 | 5.95E-06 |
| GO:0030182~neuron differentiation | 1.75 | 3.61E-09 | 6.74E-06 |
| GO:0032989~cellular component morphogenesis | 1.79 | 9.40E-09 | 1.75E-05 |
| GO:0000902~cell morphogenesis | 1.83 | 1.06E-08 | 1.98E-05 |
| GO:0048812~neuron projection morphogenesis | 2.07 | 1.35E-08 | 2.52E-05 |
| GO:0065007~biological regulation | 1.12 | 1.43E-08 | 2.67E-05 |
| GO:0048519~negative regulation of biological process | 1.33 | 1.50E-08 | 2.81E-05 |
| GO:0007420~brain development | 1.91 | 1.91E-08 | 3.57E-05 |
| GO:0051239~regulation of multicellular organismal process | 1.50 | 1.96E-08 | 3.65E-05 |
| GO:0048666~neuron development | 1.82 | 1.97E-08 | 3.68E-05 |
| GO:0048468~cell development | 1.59 | 1.98E-08 | 3.70E-05 |
| GO:0032990~cell part morphogenesis | 1.96 | 2.15E-08 | 4.02E-05 |
| GO:0050789~regulation of biological process | 1.13 | 2.39E-08 | 4.45E-05 |
| GO:0048568~embryonic organ development | 2.19 | 2.44E-08 | 4.55E-05 |
| GO:0048523~negative regulation of cellular process | 1.34 | 2.62E-08 | 4.89E-05 |
| GO:0048858~cell projection morphogenesis | 1.97 | 2.83E-08 | 5.28E-05 |
| GO:0007409~axonogenesis | 2.09 | 3.78E-08 | 7.05E-05 |
| GO:0050794~regulation of cellular process | 1.13 | 4.86E-08 | 9.07E-05 |
| GO:0048522~positive regulation of cellular process | 1.31 | 6.91E-08 | 1.29E-04 |
| GO:0048667~cell morphogenesis involved in neuron differentiation | 2.00 | 1.22E-07 | 2.27E-04 |
| GO:0000904~cell morphogenesis involved in differentiation | 1.91 | 1.92E-07 | 3.58E-04 |
| GO:0006357~regulation of transcription from RNA polymerase II promoter | 1.50 | 2.01E-07 | 3.75E-04 |
| GO:0048562~embryonic organ morphogenesis | 2.28 | 2.32E-07 | 4.33E-04 |
| GO:0031175~neuron projection development | 1.87 | 2.41E-07 | 4.49E-04 |
| GO:0009891~positive regulation of biosynthetic process | 1.52 | 2.82E-07 | 5.27E-04 |
| GO:0045941~positive regulation of transcription | 1.57 | 3.54E-07 | 6.61E-04 |
| GO:0030900~forebrain development | 2.18 | 3.56E-07 | 6.64E-04 |
| GO:0009887~organ morphogenesis | 1.58 | 3.70E-07 | 6.90E-04 |
| GO:0048518~positive regulation of biological process | 1.28 | 4.25E-07 | 7.93E-04 |
| GO:0010557~positive regulation of macromolecule biosynthetic process | 1.52 | 6.57E-07 | 0.00122603 |
| GO:0010646~regulation of cell communication | 1.40 | 7.19E-07 | 0.001342576 |
| GO:0010628~positive regulation of gene expression | 1.55 | 7.25E-07 | 0.001353424 |
| GO:0031328~positive regulation of cellular biosynthetic process | 1.51 | 7.33E-07 | 0.001367361 |
| GO:0010629~negative regulation of gene expression | 1.58 | 9.28E-07 | 0.001731976 |
| GO:0051173~positive regulation of nitrogen compound metabolic process | 1.50 | 1.46E-06 | 0.002724958 |
| GO:0045893~positive regulation of transcription, DNA-dependent | 1.59 | 1.47E-06 | 0.002735768 |
| GO:0016481~negative regulation of transcription | 1.60 | 1.58E-06 | 0.002944839 |
| GO:0051254~positive regulation of RNA metabolic process | 1.58 | 2.31E-06 | 0.004305911 |
| GO:0045935~positive regulation of nucleobase, nucleoside, nucleotide and nucleic acid metabolic process | 1.49 | 2.88E-06 | 0.005372248 |
| GO:0048598~embryonic morphogenesis | 1.71 | 3.19E-06 | 0.005961884 |
| GO:0048732~gland development | 2.16 | 3.32E-06 | 0.006198934 |
| GO:0007389~pattern specification process | 1.76 | 3.98E-06 | 0.007433438 |
| GO:0030030~cell projection organization | 1.63 | 5.38E-06 | 0.01004259 |
| GO:0006355~regulation of transcription, DNA-dependent | 1.27 | 5.54E-06 | 0.010343551 |
| GO:0045944~positive regulation of transcription from RNA polymerase II promoter | 1.63 | 8.50E-06 | 0.015857123 |
| GO:0051172~negative regulation of nitrogen compound metabolic process | 1.50 | 1.53E-05 | 0.028487024 |
| GO:0045934~negative regulation of nucleobase, nucleoside, nucleotide and nucleic acid metabolic process | 1.50 | 1.61E-05 | 0.030110173 |
| GO:0051252~regulation of RNA metabolic process | 1.25 | 1.82E-05 | 0.033934227 |
| GO:0009966~regulation of signal transduction | 1.38 | 1.89E-05 | 0.035279814 |
| GO:0009790~embryonic development | 1.46 | 2.04E-05 | 0.038053731 |
| GO:0050793~regulation of developmental process | 1.43 | 2.13E-05 | 0.039805895 |
| GO:0045892~negative regulation of transcription, DNA-dependent | 1.60 | 2.15E-05 | 0.040039978 |
| GO:0051253~negative regulation of RNA metabolic process | 1.59 | 2.32E-05 | 0.043308227 |
