# Supplementary material for: Genome-Wide DNA Methylation Scan in Major Depressive Disorder
Source: PLoS One. 2012 Apr 12;7(4):e34451. doi: 10.1371/journal.pone.0034451 (PMC3325245; doi:10.1371/journal.pone.0034451)
Supplement: Table S3 — Bisulfite pyrosequencing was used to experimentally validate some of the regions that showed differential methylation between MDD and controls by CHARM analysis. This table shows those that were nominally validated. P-values for regression of pyrosequencing methylation at individual CpGs (rows) on 6 covariates (columns). The last column shows the F-statistic p-value for the multiple regression of methylation on all 6 covariates. (DOC) [file pone.0034451.s004.doc]

| **Table S3: Impact of demographic, clinical, and biologic variables on nominally significant DNAm differences between MDD and controls** | | | | | | | | | |
| --- | --- | --- | --- | --- | --- | --- | --- | --- | --- |
| CpG | Control mean DNAm % | MDD mean DNAm % | Axis I primary dx | Age | Sex | Left brain | Smoking at TOD | Lifetime alcohol use | F |
| *CPSF3* (1) | 58.0 | 65.0 | 0.00099 | 0.31 | 0.22 | 0.89 | 1.00 | 0.31 | 0.023 |
| *CPSF3* (2) | 66.5 | 70.8 | 0.0019 | 0.89 | 0.44 | 0.31 | 0.19 | 0.78 | 0.088 |
| *EEPD1* | 74.1 | 58.8 | 0.041 | 0.56 | 0.67 | 0.72 | 0.79 | 0.15 | 0.52 |
| *FAM107B* | 75.7 | 80.5 | 0.013 | 0.0041 | 0.54 | 0.67 | 0.37 | 0.26 | 0.062 |
| *ISOC1* | 92.3 | 91.3 | 0.045 | 0.40 | 0.47 | 0.16 | 0.88 | 0.0041 | 0.057 |
| *KIAA0100* | 71.5 | 73.9 | 0.033 | 0.0065 | 0.52 | 0.65 | 0.51 | 0.20 | 0.038 |
| *LASS2* (3) | 91.7 | 93.2 | 0.0034 | 0.12 | 0.73 | 1.00 | 0.91 | 0.80 | 0.15 |
| *LASS2* (5) | 87.6 | 91.2 | 0.032 | 0.38 | 0.60 | 0.48 | 0.29 | 0.38 | 0.14 |
| *LASS2* (6) | 91.2 | 92.1 | 0.040 | 0.20 | 0.67 | 0.45 | 0.75 | 0.061 | 0.18 |
| *LASS2* (7) | 93.3 | 94.3 | 0.0024 | 0.35 | 0.58 | 0.80 | 0.66 | 0.037 | 0.037 |
| *PLCE1* | 69.0 | 72.2 | 0.023 | 0.64 | 0.24 | 0.85 | 0.30 | 0.085 | 0.079 |
| *PRIMA1* (1) | 40.9 | 51.9 | 0.0062 | 0.053 | 0.57 | 0.99 | 0.30 | 0.74 | 0.17 |
| *PRIMA1* (2) | 44.7 | 59.1 | 0.00027 | 0.044 | 0.88 | 0.90 | 0.51 | 0.71 | 0.043 |
| *PRIMA1* (3) | 53.4 | 67.9 | 0.00028 | 0.063 | 0.64 | 0.72 | 0.58 | 0.55 | 0.024 |
| *PRIMA1* (4) | 58.4 | 72.2 | 0.00026 | 0.083 | 0.85 | 0.80 | 0.52 | 0.60 | 0.036 |
| *PRIMA1* (5) | 65.6 | 77.1 | 0.00019 | 0.072 | 0.95 | 0.96 | 0.70 | 0.46 | 0.028 |
| *ZNF263* (1) | 39.1 | 45.8 | 0.0061 | 0.054 | 0.63 | 0.38 | 0.20 | 0.29 | 0.053 |
| *ZNF263* (2) | 42.9 | 51.3 | 0.0027 | 0.016 | 0.68 | 0.32 | 0.19 | 0.24 | 0.023 |
| *ZNF263* (3) | 47.9 | 55.2 | 0.0048 | 0.064 | 0.35 | 0.37 | 0.51 | 0.19 | 0.036 |
| *ZSCAN29* | 72.4 | 88.5 | 0.011 | 0.23 | 0.14 | 0.061 | 0.89 | 0.84 | 0.13 |
