# Supplementary material for: Genome-Wide DNA Methylation Scan in Major Depressive Disorder
Source: PLoS One. 2012 Apr 12;7(4):e34451. doi: 10.1371/journal.pone.0034451 (PMC3325245; doi:10.1371/journal.pone.0034451)
Supplement: Supporting Information S1 — Supplementary Tables S4, S5, S6 show results of replication attempts in postmortem brain and in lymphoblastoid cell lines. (DOCX) [file pone.0034451.s005.docx]

| **Table S4: *PRIMA1* DNAm in replication set of 16 MDD postmortem brain samples and 13 controls** | | | |
| --- | --- | --- | --- |
| CpG | Control mean DNAm % | MDD mean DNAm % | P-value |
| *PRIMA1* (1) | 55.7 | 52.5 | 0.18 |
| *PRIMA1* (2) | 62.9 | 59.6 | 0.20 |
| *PRIMA1* (3) | 76.3 | 71.9 | 0.13 |
| *PRIMA1* (4) | 76.8 | 73.6 | 0.25 |
| *PRIMA1* (5) | 81.5 | 78.8 | 0.19 |

| **Table S5: DNAm in postmortem brain replication set *(CPSF3*, *LASS2*, *ZNF263*)** | | | |
| --- | --- | --- | --- |
| CpG | Control mean DNAm % | MDD mean DNAm % | P-value |
| *CPSF3* (1) | 69.6 | 66.5 | 0.20 |
| *CPSF3* (2) | 73.9 | 71.6 | 0.11 |
| *LASS2* (1) | 93.0 | 92.8 | 0.50 |
| *LASS2* (2) | 89.6 | 89.5 | 0.94 |
| *LASS2* (3) | 91.2 | 91.3 | 0.66 |
| *LASS2* (4) | 93.9 | 92.1 | 0.45 |
| *ZNF263* (1) | 57.3 | 53.7 | 0.32 |
| *ZNF263* (2) | 61.1 | 57.7 | 0.36 |
| *ZNF263* (3) | 64.5 | 62.0 | 0.43 |

| **Table S6: *PRIMA1* DNAm in a lymphoblastoid cell line replication set (N=90 cases and 90 controls)** | | | |
| --- | --- | --- | --- |
| CpG | Control mean DNAm % | MDD mean DNAm % | P-value |
| *PRIMA1* (1) | 46.6 | 45.2 | 0.48 |
| *PRIMA1* (2) | 37.5 | 38.2 | 0.69 |
| *PRIMA1* (3) | 69.5 | 68.5 | 0.57 |
| *PRIMA1* (4) | 51.7 | 52.1 | 0.79 |
| *PRIMA1* (5) | 61.1 | 58.7 | 0.18 |
